# Simultaneous Dynamic Light Scattering, Absorbance and Photoluminescence Measurements of Colloidal Nanoparticles. Application to Colloidal Stability and Aggregation Kinetics of CsPbBr_3_ Nanocrystals

**DOI:** 10.1002/smtd.202500304

**Published:** 2025-10-30

**Authors:** Pietro Anzini, Maria Chiara Bossuto, Mara Colombo, Anna Vivani, Ihor Cherniukh, Maryna I. Bodnarchuk, Maksym V. Kovalenko, Federica Bertolotti, Antonietta Guagliardi, Norberto Masciocchi, Fabio Ferri

**Affiliations:** ^1^ Dipartimento di Scienza e Alta Tecnologia and To.Sca.Lab Universita degli Studi dell'Insubria Via Valleggio 11 Como I‐22100 Italy; ^2^ CLIP‐Como Lake Institute of Photonics via Valleggio 11 Como 22100 Italy; ^3^ Department of Chemistry and Applied Bioscience Institute of Inorganic Chemistry ETH Zurich, Vladimir Prelog Weg 1 Zurich CH‐8093 Switzerland; ^4^ Laboratory for Thin Films and Photovoltaics Empa—Swiss Federal Laboratories for Materials Science and Technology Dubendorf CH‐8600 Switzerland; ^5^ Istituto di Cristallografia (IC) and To.Sca.Lab Consiglio Nazionale delle Ricerche (CNR) via Valleggio 11 Como I‐22100 Italy

**Keywords:** absorbance, colloidal nanoparticles, dynamic light scattering, perovskites nanocrystals, photoluminescence

## Abstract

An original but simple instrument is developed, capable of performing simultaneous Dynamic Light Scattering (DLS), Absorbance (ABS), and Photoluminescence (PL) measurements on suspensions of colloidal nanoparticles. The instrument is designed as a standard laboratory tool that, by using only commercial components, enables rapid analysis of the sample under investigation. The setup offers two significant benefits: first, it allows for the simultaneous analysis of the very same sample, overcoming limitations arising from the measurements being performed on distinct specimens, under diverse experimental settings, at different times; second, it enables to monitor the kinetic evolution of nanocrystals (Ncs) colloidal stability in time, with a time resolution of ≈ 100 s, which is considerably shorter than typical timescales related to aggregation kinetics or other colloidal instability‐related phenomena. The suitability of the instrument for characterizing time‐resolved processes is assessed through the case study of the long‐term instability of oleic acid/oleylamine‐capped colloidal CsPbBr_3_ NCs suspensions driven by dilution. It is found that, as ligands gradually desorb from the surface, the suspension becomes unstable, displaying two rather unexpected processes: the formation of transient randomly arranged NCs aggregates that reach micron‐sized dimensions, followed by the coalescence of the original NCs into larger crystalline domains.

## Graphical abstract

An original but simple instrument capable of performing simultaneous Dynamic Light Scattering, Absorbance and Photoluminescence measurements on suspensions of colloidal nanocrystals (NCs) is realized as a standard laboratory tool. It enables to easily monitor the NCs colloidal stability or to characterize their aggregation kinetics or other colloidal instability‐related phenomena in a time resolved manner, with a 100 s time resolution.

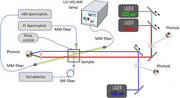

## 1 Introduction

Semiconductor nanocrystals (NCs) with size‐tunable optical and electronic properties represent a new generation of low‐cost solution‐processable materials, prepared by well‐established synthetic protocols for a wide spectrum of technological applications, from photovoltaics to lighting.^[^ ^1^, ^2^, ^3^, ^4^, ^5^ ^]^ NCs can be produced by using different methods,^[^ ^6^ ^]^ but, undoubtedly, the colloidal synthesis is presently one of the most effective at obtaining ligands‐stabilized NCs that are highly uniform in size and shape. In this context, following the pioneering work done by the Kovalenko's group^[^ ^7^ ^]^ in 2015 on nanosized cesium lead halide perovskites, the field of colloidal metal (mostly lead‐based) halide perovskite (LHP) NCs, has recently experienced a steady and rapid expansion (exceeding that of classical II‐VI and III‐V quantum dots), driven by their superior optoelectronic properties, simple preparation, and the wider spectrum of technological applications, extending beyond those mentioned above.^[^ ^8^, ^9^ ^]^ Despite such an exciting scenario, the colloidal stability of LHP NCs remains a more challenging task than that of classical quantum dots. One major issue is due to the ligand desorption, particularly when common ligands (carboxylic acids and primary or secondary amines, such as ubiquitous oleic acid (OAc) and oleylamine (OAm)), are used. Recent advances in this direction have been obtained that rely on tight binding long‐chain zwitterionic ligands (retained upon several purification cycles) or post‐synthetic replacement procedures.^[^ ^10^, ^11^ ^]^

Therefore, the characterization of LHP and of other classes of semiconductor NCs in their colloidal state is of paramount importance. This requires fast and efficient experimental/analytical tools for assessing fundamental properties such as size, shape, dispersion and stability upon dilution/concentration, aggregation/self‐assembly, ligands desorption and particles degradation. All these aspects have relevant implications in the optimization of synthetic protocols at the lab scale, to govern the scale‐up and manufacturing pipelines and, ultimately, to determine the performance of the final dry NCs integrated into optoelectronic devices.^[^ ^12^ ^]^

As said, crucial initial steps are probing, at the lab scale in a fast manner, both the NCs size and their spectroscopic properties and, after that, easily monitoring the colloidal stability. In this regard, absorbance and emission spectra are rapidly measured on highly diluted suspensions of NCs using commercially available and efficient spectrophotometers covering the UV–vis‐NIR range. The bottleneck, slowing down the entire process, is the acquisition of a reliable measure of the NC size and its mono/polydispersity through lengthy transmission electron microscopy (TEM) measurements, which cannot be acquired in the same fast manner for most of the nanomaterials of technological interest. Practical considerations have suggested to overcome such an issue by constructing the so‐called sizing curves, relating the (size‐dependent) band edge transition energies to the NC sizes over a reasonable range of values. Thus, by using these empirical relationships, the size of freshly prepared NC colloids can be indirectly estimated from their quickly measured absorbance/emission spectra.

However, although TEM measurements used for constructing these sizing curves analyze individual particles and therefore provide a direct measurement of their sizes and shape distributions (from 2D projections), they suffer from some shortcomings such as: a) the statistical robustness of the results is rather poor since the analysis typically covers only a few hundreds of NCs; b) the boundaries of very small NCs are often structurally disordered and are smeared out in low‐contrast images; c) the NC samples must be treated prior to measurement (in high vacuum environments) and might be unstable under the electron beam (as commonly observed in soft LHP NCs). These shortcomings hamper the efficiency of the TEM analysis [points (a) and (b)] and can further induce significant alteration of NCs structure and morphology [point (c)].^[^ ^13^ ^]^

Small Angle X‐ray Scattering (SAXS) techniques have recently emerged as a viable alternative to TEM for sizing curves construction, overcoming the intrinsic limitations of imaging analysis.^[^ ^13^ ^]^ Indeed, SAXS works directly on colloidal suspensions and statistics is very robust because of the very large number of NCs (higher than 10^12^) simultaneously probed. On the other hand, data modeling requires some expertise and data analysis may not be straightforward. Recent studies have also analyzed the many TEM‐ and SAXS‐based sizing curves presently available for the most important classes of quantum dots/NCs, including metal chalcogenides and pnictides and LHPs.^[^ ^14^ ^]^ Significant discrepancies are found within the same class of NCs, attributed to multiple potential sources of technical or human origin in measurement/data analysis, affecting both size and band edge energy measurements.^[^ ^14^ ^]^

Similar to SAXS, other scattering techniques can analyze data collected on NCs colloidal suspensions and offer similar benefits, but they are less common for size analysis. These are: Dynamic Light Scattering (DLS),^[^ ^15^, ^16^ ^]^ Static Light Scattering (SLS),^[^ ^17^, ^18^ ^]^ and X‐ray Powder Diffraction (XRPD) through the Wide Angle X‐ray Total Scattering (WAXTS) methods both in the reciprocal space^[^ ^19^, ^20^, ^21^ ^]^ and direct space domains.^[^ ^22^, ^23^, ^24^ ^]^ For the interested reader, an extended overview of spectroscopic and scattering methods that can be used for a comprehensive characterization of NCs in terms of composition, structure, size and shape distributions, is presented.^[^ ^25^ ^]^

From a general point of view, the use of multiple techniques is highly recommended and offers a great advantage for a thorough characterization of colloidal NCs because they provide complementary information and probe different length scales. Nonetheless, there are shortcomings arising from separate measurements performed on distinct specimens under different experimental conditions (often optimized for the specific technique, for example requiring diluted or concentrated samples), at different times. This is a common problem associated with the available experimental sizing curves, which are typically obtained from physically distinct specimens. Furthermore, some of the required instrumentation may not be available in a standard laboratory (this may be the case of SAXS instruments) or necessitate high‐power radiation sources that are typically available only at large‐scale facilities, for example synchrotron‐based WAXTS methods which perform well on rather concentrated samples (≈ 5 mg mL^−1^ and above).

In this work, we present the development of a simple instrument that, by using only commercial components, allows simultaneous DLS, Absorbance (ABS) and Photoluminescence (PL) measurements to be carried out. We believe that integrating non‐invasive light scattering and spectroscopic methods into a single setup offers two major advantages: i) it allows to simultaneously measure the very same sample (i.e., simultaneously within the same optical cell), thereby overcoming key limitations of standard lab practices for NCs sizing; ii) when operated in a time‐resolved modality, it allows the kinetic evolution of the colloidal stability of NCs to be monitored. While the coupling of different techniques to characterize colloidal NCs is nowadays commonly implemented at large‐scale facilities, SAXS‐WAXTS, SAXS‐DLS, and SAXS or WAXTS coupled to ABS/PL spectroscopy being the most popular ones,^[^ ^26^, ^27^, ^28^, ^29^ ^]^ the proposed instrument is intentionally conceived as *a standard laboratory tool*. It provides a fast analytical solution capable of acquiring all the three signals within ≈ 100 s and monitoring NCs stability over a desired timescale.

The following sections deal with experimental and theoretical aspects of the proposed setup, discuss DLS/ABS/PL measurements simultaneously extracted from simple experiments using CsPbBr_3_ colloidal NCs as benchmark samples, and compare the output parameters to those from available sizing curves. These results are complemented by a case study on the time‐evolution of CsPbBr_3_ colloids,^[^ ^30^ ^]^ prone to aggregation and degradation upon dilution and storage for a prolonged time, with rather unexpected outcomes.

## 2 Experimental Section

**Figure 1:**
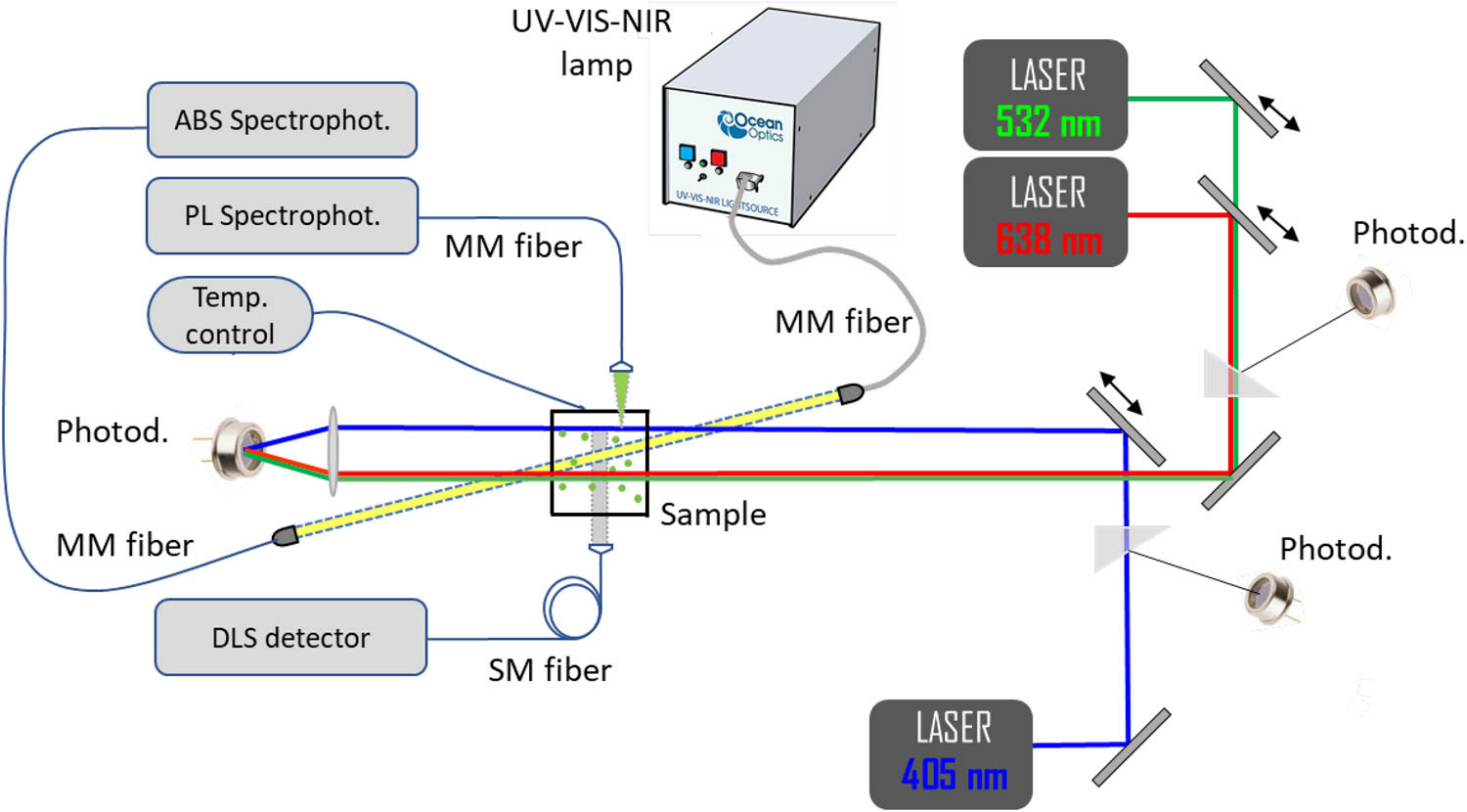
Scheme of the entire instrument built for simultaneous DLS, ABS and PL measurements on colloidal NCs. A top‐view photo of the actual instrument is showcased in the, Figure S9 Supporting Information.

The experimental apparatus, which allows quasi‐simultaneous measurements of DLS, ABS and PL, is sketched in **Figure** **1**. It includes a UV–VIS‐IR (white) source lamp (Ocean Insights, mod DH2000‐BAL), two fiber‐optic spectrophotometers for ABS and steady–state PL excited by a tunable power 405 nm CW laser (Integrated Optics, model 0405‐21A‐NI‐NT‐CF, λ = 404.9 nm, *P* = 53 mW) plus a home‐made DLS apparatus that collects the light scattered typically at 90°, but can also be operated at different scattering angles. ABS and PL measurements are acquired using two spectrophotometers (Ocean Optics, mod HR2000‐GC‐UV‐NIR) operating in the UV–VIS‐NIR 200–1100 nm range, connected to multi‐mode (MM) fibers with cores between 200 and 600 µm. Both spectrophotometers are housed in a thermalized box, so to increase their long‐term stability.

DLS measurements are taken by collecting the scattered light with a single‐mode (SM) fiber. Depending on the sample being studied, different laser sources can be employed: a green laser (Coherent, model COMPASS 315M‐100, λ = 532 nm, *P* = 100 mW), or a red laser (Integrated Optics, 638 nm SLM Laser, 0638L‐25A‐NI‐AT‐NF with PM fiber, λ = 637.8 nm, *P* = 60 mW) or the blue laser described above. The output of the fiber was coupled to a commercial single‐photon “pseudo” cross‐correlation detector (ALV, model ALV/SO‐SIPD) for eliminating detector defects (after‐pulses and dead time). Data were acquired with a fast commercial counter board and the DLS analysis (i.e., the computation of the correlation function) was carried out either in real‐time or off‐line via a software correlator.^[^ ^30^ ^]^ This flexible data acquisition scheme offers the possibility of saving the raw data, post‐processing them, and performing a dedicated data reduction for dust discrimination and time‐resolved batch analysis.^[^ ^31^ ^]^ This option is of vital importance for getting reliable DLS results because NCs colloidal suspensions are often contaminated with ineliminable impurities that strongly distort the correlation function and impair the DLS analysis (as it may frequently observed in commercial DLS particle sizers).^[^ ^32^ ^]^

Although, in principle, DLS, ABS and PL measurements could be taken simultaneously, for practical reasons related to stray‐light rejection, they are most usefully carried out sequentially, with the white source lamp, the 405 nm laser, and the 532 nm (or 638 nm) laser selected one at a time. The switching between DLS, ABS and PLmeasurements takes place quite rapidly, by inserting or removing the various movable optical mirrors placed along the optical axis using a remote electronic control, a task that requires only a fraction of a second. Considering that the typical duration of ABS and PL measurements were $\Delta t_{\mathrm{ABS}}\approx \Delta t_{\mathrm{PL}}\approx 5-20\;s$ whereas for DLS $\Delta t_{\mathrm{DLS}}\approx 50-100\;s,$ the whole measurement lasts $\Delta t_{\mathrm{DLS}}+\Delta t_{\mathrm{ABS}}+\Delta t_{\mathrm{PL}}\approx 100\;s$. Such a time duration was remarkably shorter than the time scales over which the aggregation kinetics or other instabilities of colloidal NCs develop. In particular, for the experiments later discussed, such time scales are of the order of hours or days, implying that, as a matter of fact, the measurements carried out with the instrument described in this work were practically simultaneous.

The sample was contained in a standard quartz‐glass square cuvette, which was jacketed by a temperature‐controlled holder mounted on a *xyz* translation stage. Thus, the sample can be moved longitudinally and transversally to the optical axis, allowing control and optimization of the optical path of the radiation inside the sample. Such information was crucial for properly correcting the PL data for self‐absorption effects. Three photodiodes monitor the incident and transmitted power of the laser light, enabling a proper normalization of the PL spectra and the measurement of the sample attenuation. Finally, all the mechanical, optical and electro‐optical components of this prototype were fully remotely controlled and tuned by a personal computer.

This Section ends by discussing the limitations imposed on this setup by the simultaneous measurements of DLS, ABS and, PL in terms of sample concentration requirements. Indeed, considering that the sample was contained in a 1 cm thick quartz cuvette, the appropriate concentration levels for the three measurements might be different. In this regard, ABS appears to be the most lenient technique. Indeed, these spectrophotometers can accurately measure ABS value in the range ≈ 0.01 − 2, which corresponds to a dynamic range of slightly more than two decades in sample concentration [because of the linearity between concentration and ABS, see Equation (8)]. Thus, the ABS of both highly absorbing and transparent samples can be readily determined with this setup. In the case of very concentrated samples, thinner cuvettes can be accommodated in the sample holder. As to PL measurements, the concentration requirements depend on various factors, such as the sensitivity of the spectrophotometer detector and its optical configuration, the quantum yield of the photo luminescent particles, the wavelength and the power of the exciting laser, and the solid angle of the collection optics. For fluorescent nanoparticles with high quantum yield, PL measurements can be easily performed with this setup across a broad range of sample concentrations. The only precaution to take was minimizing self‐absorption contributions, which can be accounted for and corrected by making use of Equation (10). The most critical technique of this setup was DLS because, when the nanoparticles were in the range of ≈ 10 nm (or smaller), their scattering signal was fairly weak and, correspondingly, the DLS data were very noisy. This is an intrinsic limitation of the DLS technique, which could be tackled either by using better hardware components (more powerful lasers or detectors with higher quantum efficiency) or by increasing sample concentration and taking longer measurements. (for a quantitative analysis of the noise associated to DLS and corresponding detection limits see Section 9). However, sample concentration cannot be increased excessively, as this would invalidate the assumptions of non‐interacting particles and single scattering (see Section 6.1). Additionally, also PL self‐absorption effects would become non‐negligible (see Section 6.3). On the other hand, the increase of the measuring time leads to a subsequent reduction of the time resolution of the setup.

Summarizing, quantifying a range of sample concentrations appropriate for all three techniques was not straightforward, but was bottlenecked by the limitations of the DLS method. For stable NC samples (as those described in the next Sections S3 and S6, Supporting Information) where, in principle there were no limits on measuring times, DLS (as well as ABS and PL) can be performed on ≈ 10 nm NCs at concentrations as small as ≈ 0.01 mg mL^−1^, with the possibility of increasing the concentration up to ≈ 0.5 mg mL^−1^ without jeopardizing the reliability of any of the three techniques. For evolving samples, a tradeoff between time resolution and accuracy of DLS data was unavoidable, as shown in the case study reported in Section 4.

## 3 Sizing of CsPbBr_3_ Perovskites NCs

As a paradigmatic example of the use of our instrument for the characterization of NC suspensions, we report the case of three CsPbBr_3_ perovskites samples (labelled as LHP‐A, LHP‐B, and LHP‐C in **Table** **1**) that were synthesized as ligand‐stabilized colloidal NCs with sizes of ≈7−8nm (from TEM, see Supporting Information) at a nominal concentration of 7 mg mL^−1^ in toluene (for details on the sample synthesis see Section S1, Supporting Information). The capping ligands are used to passivate the NCs surface and stabilize the suspension inducing steric repulsion between the NCs.^[^ ^33^ ^]^ The ligands used in our CsPbBr_3_ NCs are oleic acid and oleylamine (OAc/OAm).

**Table 1:** Comparison between DLS, ABS, PL and TEM sizing techniques.

sample (7 mg/mL)	dilution	conc [µg/mL]	DLS		TEM			ABS		PL	
			$d_{DLS}$ [nm]	$L_{DLS}$ [nm]	${\left\langle \ell_{\mathrm{TEM}}\right\rangle }_{n}$ [nm]	${\left\langle L_{\mathrm{TEM}}\right\rangle }_{z}$ [nm]	$\left\langle \alpha_{\mathrm{TEM}}\right\rangle$ [nm]	$E_{g}$ [nm]	$\ell_{\mathrm{ABS}}$ [nm]	$\lambda_{\mathrm{peak}}$ [nm]	[FWHM] [nm]
									ref. [36]		
LHP‐A	1:50	140	13.3	**10.0**	*7.5*	**10.3**	1.3	2.473	*7.4*	507.5	19.7
LHP‐B	1:100	70	13.5	**10.2**	*7.5*	**10.1**	1.1	2.463	*7.8*	509.5	18.3
LHP‐C	1:50	140	14.1	**10.7**	*8.1*	**10.8**	1.2	2.461	*7.9*	510.3	18.7

**Figure 2:**
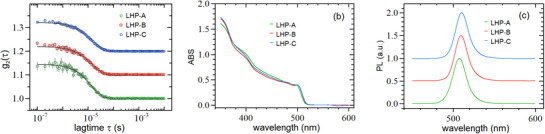
DLS, ABS, and PL data for the three samples of Table 1. For the sake of clarity, in panel a) the red and blue curves have been shifted upwards, in panel b) the curves have been rescaled so to overlap and in panel c) they have been normalized to their maximum and shifted upwards.

The three samples listed in Table 1 were diluted by factors of 50 and 100, and measured with our setup repetitively every ≈0.3h as long as they did not show any sign of instability (due to NCs aggregation, see next section). The DLS measurements were carried out with the green laser at full power (≈ 100 mW); the choice of using the green laser (instead of the red laser) is justified by the fact that, in the case of our particular setup, it provides (at equal measuring time) a better signal to noise ratio of the correlation data (see Section S6, Supporting Information). The PL measurements were taken by exciting the sample with the blue laser at rather low power (≈2 mW), so to avoid sample photo‐bleaching and sample damage. In order to reduce the noise (mainly of the DLS data), the various measurements were averaged, and the results are showcased in the three panels of **Figure** **2**, which reports the average correlation functions (*g*_2_, Figure 2a), average absorbance spectra (ABS, Figure 2b), and average photoluminescence spectra (PL, Figure 2c). The results of the data analysis based on the fitting and numerical procedures described in the theoretical section (Section 6, where all equations are detailed), are summarized in Table 1, where we also report the findings of the TEM analysis performed on the same colloidal NCs as described in the Section S2 (Supporting Information). The edge length $\ell_{\mathrm{ABS}}$ obtained from ABS sizing curves are also quoted for an extended discussion.

**Figure 3:**
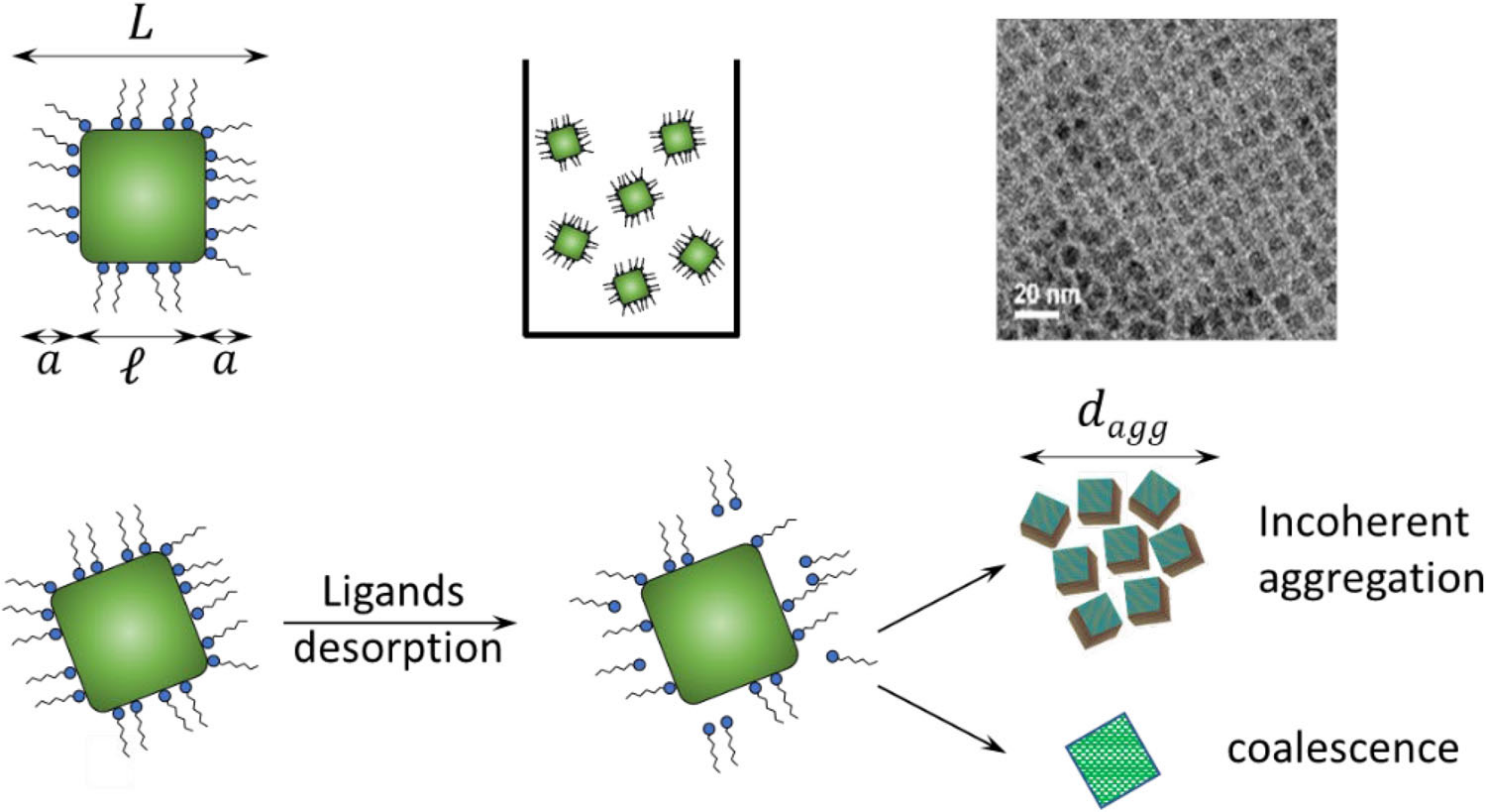
Proposed mechanism of NCs instability derived from the analyses of the DLS, ABS and PL data discussed below. The ligands desorption induced by dilution may lead to the formation of incoherent aggregates or larger coherent domains where the original NCs are fused together (coalescence). Note that the aggregates and the coalesced particle are not to scale with the pristine NC portrayed in the leftmost portion of the figure.

For a quantitative comparison between the various techniques, we assume that the CsPbBr_3_ NCs have a nearly cuboidal shape^[^ ^34^, ^35^ ^]^ and, as sketched in **Figure** **3**, we indicate with ℓ the edge length of the NC core and with *a* the ligand length; as a consequence, the edge length *L* of the whole jacketed (core + shell) NCs is L=ℓ+2a.

For the analysis of the DLS data, we recall that this technique probes the so‐called hydrodynamic diameter *d*_DLS_ of the NCs (see Theoretical Section 6 for details), a value that also includes the hydrodynamic hindering from the long ligand chains attached to the NCs surface. Thus, the ligands behave as a shell that reduces the NCs diffusivity, often in an unpredictable manner since they are flexible, and the solvent can flow through them during the NCs Brownian motion. In addition, we note that, as far as DLS is concerned, these samples can be considered *practically* monodisperse because even a 10% polydispersity (as estimated from TEM analysis, see Supporting Information) is hardly detectable with this technique.^[^ ^18^ ^]^ Furthermore, as detailed in the theoretical Section 6.1, the NCs fluorescence generates a high background signal that dampens the amplitude (β_obs_) of the correlation function (*g*_2_) to values significantly smaller than unity [see Equation (3)], making the DLS estimate of the NCs size not straightforward. To overcome these difficulties, the DLS data were fitted to a single exponential decay function [Equation (3)] obtaining rather similar estimates of the hydrodynamic diameter, i.e., $d_{\mathrm{DLS}}\approx 13-14\;\mathrm{nm}$. By assuming a cubic shape for the NCs, we used Equation (5) for estimating the edge Lengths *L*_DLS_ of the jacketed NCs, obtaining $L_{\mathrm{DLS}}\approx 10-11\;\mathrm{nm}$. Finally, we quantified the relative strengths between scattering and fluorescence signals (see Section S5, Supporting Information). Our findings show that, for all three samples of Table 1, ≈40% of the (dark subtracted) signal is due to scattering, whereas the remaining 60% is due to fluorescence.

For comparison, we report in Table 1 the estimates of the NC n and z‐average edge lengths ${\left\langle \ell_{\mathrm{TEM}}\right\rangle }_{n}$ and 〈*L*_TEM_〉_*z*_, together with the average ligand length 〈*a*_TEM_〉 as extracted from the analysis of TEM images. As detailed in the Section S2 (Supporting Information), ${\left\langle \ell_{\mathrm{TEM}}\right\rangle }_{n}$ was determined by standard statistical analysis of the (segmented and filtered) images, whereas  〈*L*_TEM_〉_*z*_ was determined as the average distance between the first NC neighbors. Thus,  〈*L*_TEM_〉_*z*_ is the size parameter that compares directly to *L*_DLS_ and the data of Table 1 (bold fonts) show that the two techniques provide quite consistent results.

From the ABS measurements, we estimated the energy gap *E_g_* of the first excitonic transition by determining the minimum of the second derivative of the ABS spectrum (see Section 6.2). The energy gap (see Table 1) can be used to estimate, via the plethora of empirical sizing curves available in literature,^[^ ^7^, ^34^, ^35^, ^36^, ^37^, ^38^ ^]^ the average edge length $\ell_{\mathrm{ABS}}$ of the NC core (see Section S3, Supporting Information). As already discussed in the introduction, the values of $\ell_{\mathrm{ABS}}$ strongly depend on the curve being used,^[^ ^14^ ^]^ which in turn is influenced by the experimental procedure and specific data‐set used for extracting the size (from TEM images or SAXS data). Conversely, there is only one sizing curve that has been determined theoretically from first principles,^[^ ^36^ ^]^ therefore being immune from these problems. The values of $\ell_{\mathrm{ABS}}$ extracted from this sizing curve compare quite nicely with ${\left\langle \ell_{\mathrm{TEM}}\right\rangle }_{n}$, as shown in Table 1 (in *italic*).

As to the PL measurements, we report in Table 1 only the peak wavelength λ_*peak*_ and the full width half maximum FWHM of the PL spectrum. To our knowledge, no reliable sizing curve associated to the photoluminescence peak has been reported in literature.

Finally, we tested successfully the reliability of our setup by comparing our DLS, ABS and PL measurements with the corresponding results obtained by using commercial instrumentation. This test was carried out on a stable CsPbBr_3_ NCs colloidal suspension prepared via a new synthesis based on the use of OLA‐PEA ligands. Details are reported in Section S7 (Supporting Information).

## 4 Colloidal (in)Stability of OAc/OAm Ligand‐Capped LHP NCs. Case Study CsPbBr_3_ NCs Suspensions upon Dilution

Once synthesis and purification/washing cycles are completed, ensuring the colloidal stability of NCs, possibly over a wide range of concentrations, is a fundamental prerequisite for any further processing and application. Recent studies on lecithin‐capped CsPbBr_3_ NCs have demonstrated the excellent stability of both ultra‐concentrated (exceeding 400 mg mL^−1^) and ultra‐diluted (a few ng/mL) suspensions, thanks to the zwitterionic nature and the tight lecithin binding.^[^ ^10^ ^]^

In contrast, OAc/OAm‐capped CsPbBr_3_ NCs were found to be stable only in a very narrow (1 to 10 mg mL^−1^) concentration range^[^ ^10^ ^]^ while, at lower concentrations, they may experience an irreversible aggregation or growth process.^[^ ^39^ ^]^ This instability arises from the gradual desorption of ligands from the surface, leading to a decreased steric shielding between NCs.^[^ ^40^ ^]^ As a result, the suspension may become unstable, causing the NCs to either form incoherent (i.e., randomly‐oriented) aggregates that can attain micro‐sized dimensions, or combine into larger coherent crystalline domains where the original NCs merge together creating larger NCs (coalescence).^[^ ^41^ ^]^

To demonstrate the applicability of the proposed simultaneous DLS, ABS, and PL techniques for studying time‐dependent processes, we present a case study focusing on the long‐term behavior of the instability of CsPbBr_3_ NCs suspensions upon dilution. Lecithin‐stabilized and (post‐synthesis) DDAB‐ treated NCs exhibit exceptional stability, both in terms of spectroscopic properties and size, regardless of the amount of dilution, for approximately one year and are not further discussed. Differently, OAc/OAm stabilized CsPbBr_3_ perovskites NCs are much less stable and prone to form aggregates. Although this instability is a well‐known phenomenon (see ref. [42] and references therein), the details of the aggregates growth process remain, quantitatively, poorly investigated. The characteristics and rate of aggregate formation largely depend on the degree of dilution. Accordingly, the case study discussed hereafter focuses on OAc/OAm capped samples only. We anticipate here that, for these samples, two different regimes of aggregation were found, that depend on the time elapsed since dilution; thus, we split the discussion into two separate sections, presented in paragraphs 4.1 and 4.2.

### 4.1 Aggregation Kinetics of OAc/OAm‐Capped CsPbBr_3_ NCs upon 1:100 Dilution: Short Times Behavior ( ≤ 68 h)

**Figure 4:**
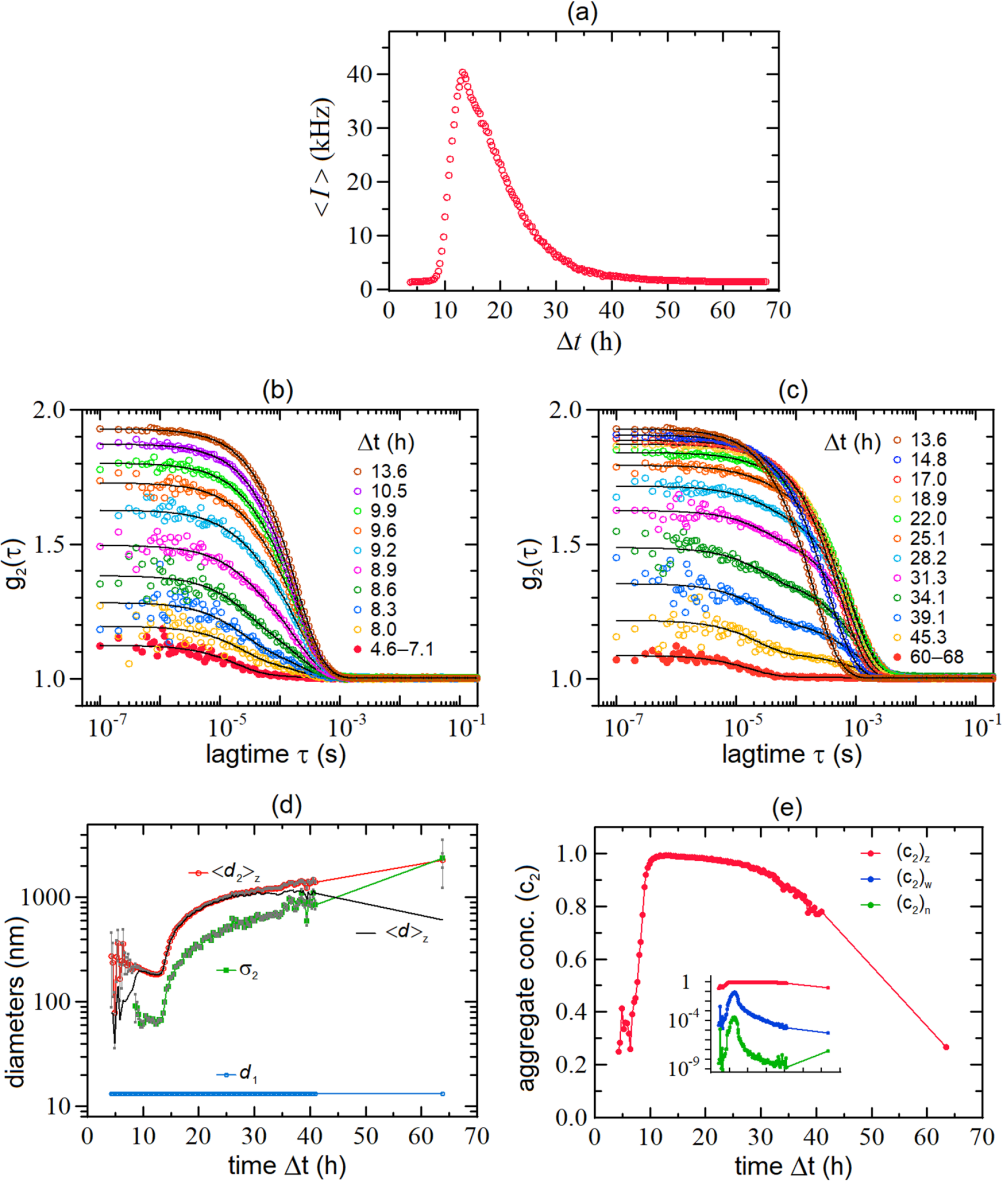
Time behavior of DLS data and fitted parameters at different times Δ*t* after dilution for an aggregating suspension of OAc/OAM‐capped CsPbBr_3_ NCs (sample LHP‐A of Table 1) diluted 1:100. a): the average Intensity 〈*I*〉 detected at 90° starts to increase after a latency time of Δ*t_lat_* ≈ 8 h reaching its maximum amplitude after $\Delta t_{\mathrm{peak}}\approx 13\;h$; thereafter, it monotonically decreases returning to its initial value after ≈ 60 h. b): correlation functions *g*_2_(τ) and corresponding fits (black curves) at times Δ*t* ≤ Δ*t_peak_*. c): correlation functions *g*_2_(τ) at times Δ*t* ≥ Δ*t_peak_*. The correlation curves shown as red solid symbols in panels (b) and (c) are averages over the indicated time intervals. d): *d*_1_ (diameter of monodisperse NCs, blue circles), 〈*d*_2_〉_*z*_ (average diameter of aggregates, red circles), $\sigma_{d_{2}}$ (standard deviation of aggregates, green squares) and 〈*d*〉_z_ = (*a*_1_ *d*_1_ + *a*_2_〈*d*_2_〉_*z*_)/(*a*_1_ + *a*_2_) (average diameter of the *overall* population, NCs and aggregates, black curve); the parameters were recovered by fitting the data of panels (b) and (c) to Equation (1). e): relative concentrations of the aggregates (*c*_2_) in terms of z‐ (red), w‐ (blue) and n‐weighted (green) fractions.

In the following, we report a representative example of the short‐times behavior (timescale of tens of hours) for the DLS, ABS and PL kinetics for a 1: 100 dilution of an OAc/OAm‐capped CsPbBr_3_ NCs suspension with an initial concentration of ≈ 7 mg mL^−1^ (sample LHP‐A of Table 1). The DLS, ABS and PL and measurements were carried out by using the same experimental settings adopted for the measurements reported in Section 3. The aggregation kinetics in this regime are well illustrated in panel (a) of **Figure** **4**, which displays the time‐dependence of the average detected intensity 〈*I*〉 detected at θ = 90° as a function of the elapsed time Δ*t* after dilution. At the very beginning, 〈*I*〉 is almost constant and rather low, i.e., ⟨I⟩≈1.5kHz. Then, only after a latency time of $\Delta t_{\mathrm{lat}}\approx 8\;h$, 〈*I*〉 starts to rapidly increase reaching a maximum amplitude ${\left\langle I\right\rangle }_{\mathrm{peak}}\approx 40\;\mathrm{kHz}$ after $\Delta t_{\mathrm{peak}}\approx 13$ h. Beyond that time, 〈*I*〉 starts to gradually decrease, returning to its initial value after ≈60h.

A similar behavior was observed at different dilutions for all the samples of Table 1, with features (Δ*t_lat_*, Δ*t_peak_*, 〈*I*〉_*peak*_) that are not completely reproducible. For example, Δ*t_lat_* may change, even at fixed dilution, by several hours, depending on sample preparation and specific sample cuvette used. This observation highlights the stochastic nature of the aggregation process, reminiscent of what is observed for the crystallization of organic species from supersaturated water solutions, where up to an eightfold increase of latency (or induction) times (at constant conditions) was observed.^[^ ^43^ ^]^ Nevertheless, we found a clear trend toward faster aggregations for more diluted samples, within the 1:50 to 1:200 dilution range.

#### 4.1.1 DLS Data

The behavior of 〈*I*〉 shown in Figure 4a is fairly consistent with the analysis of the DLS data shown in Figure 4b where the aggregation kinetics is described by the *g*_2_(τ) curves. At the very beginning (for Δ*t* < Δ*t_lat_*) the curves are quite noisy with fairly low β_obs_ amplitudes, in agreement with the low 〈*I*〉 values (see Figure 4a). It must be recalled that the detected average intensity 〈*I*〉 is the sum of two contributions, namely 〈*I*〉 =  〈*I*〉_*sca*_ + 〈*I*〉_*fluo*_, where 〈*I*〉_*sca*_ is the average scattered intensity whereas 〈*I*〉_*fluo*_ is the average fluorescence intensity. As discussed in Section S5 (Supporting Information), during these early stages, since the NC are very small ($d_{DLS}=13.3\;\mathrm{nm}$, see Table 1, sample LHP‐A), the scattering is comparable or even smaller than fluorescence [〈*I*〉_*sca*_ ≤ 〈*I*〉_*fluo*_] and, consequently, β_obs_ is very small [see Equation (3), in Section 6]. In order to reduce the noise and obtain more reliable fitting, we averaged the first 9 curves (corresponding to a time range of 2.5 h, Δ*t* = 4.6 − 7.1 h). The resulting *g*_2_(τ) curve (see first curve in Figure 4b, red solid circles) was nicely fitted by a single exponential decay function [Equation (3)], corresponding to a hydrodynamic diameter *d_h_* = 13.3 nm, which perfectly matches the initial value shown in Table 1.

Later, for Δ*t* ≥ Δ*t_lat_*, along with the increase of 〈*I*〉 portrayed in Figure 4a, the *g*_2_ curves become increasingly cleaner with β_obs_ values progressively approaching the asymptotic limit of 1. This behavior is a signature of an increase of the scattering signal, in agreement with the formation of NCs aggregates. Indeed, ${\left\langle I\right\rangle }_{sca}∼c_{\mathrm{agg}}\;d_{\mathrm{agg}}^{3}$ (where  *d*_agg_ is the size of the aggregates and *c*_agg_ their weight concentration) while 〈*I*〉_*fluo*_ remains constant, as shown below in Section 4.1.3. The *g*_2_ curves increase their amplitude without any appreciable change of decay times, suggesting that the aggregates increase their concentration *c*_agg_ without any appreciable change of *d*_agg_. This trend keeps going on until $\Delta t\approx \Delta t_{\mathrm{peak}}$, when 〈*I*〉 starts to decrease (see Figure 4a) and the *g*_2_ curves (see Figure 4c) gradually shift to the right while *reducing* their amplitudes and becoming progressively noisier. These features suggest that at $\Delta t\approx \Delta t_{\mathrm{peak}}\approx 13\;h$ there is the onset of a second aggregation step, which occurs when *c*_agg_ is high enough for the initial aggregates to interact, leading to the formation of progressively larger aggregates, up to µm scale sizes. During this second growth process, the larger aggregates inevitably start to settle at the bottom of the cuvette, provoking the progressive decrease of both 〈*I*〉 and β_obs_. This strong interplay between aggregation and sedimentation lasts for about two days, at the end of which the *g*_2_(τ) curves return quite noisy, similar to the initial ones shown in Figure 4b. However, differently from the early‐stage curves of Figure 4b with similar β_obs_’s, they deviate quite strongly from a single exponential decay, as shown by the last bottom curve of Figure 4c (red solid circles) that was obtained by averaging the last 28 curves, covering a time range of ≈8h(Δt=60−68h).

Given the rather complex kinetic behavior described above, fitting the data of Figure 4b,c was not an easy task. Based on the fact that at the beginning the solution is composed only by rather monodisperse NCs of known size (*d_h_* = 13.3 nm) and by aggregates of unknown size and distribution that form at later times, we found that the simplest function capable of fitting accurately and reliably the data was based on the presence of *only* two NCs populations: the first one representing the pristine individual NCs (“monomers”) and the second one consisting of aggregates of these monomers distributed according to a *z* − LogNormal distribution. The corresponding fitting function reads:

(1) $$g_{2}\left(q,\tau \right)=B+{\left[a_{1}e^{-\tau /\tau_{1}}+a_{2}\int_{0}^{\infty }\mathrm{LogN}\left[\tau_{2},{\langle \tau_{2}\rangle }_{z},\sigma_{\tau_{2}}\right]\;e^{-\tau /\tau_{2}}\;d\tau_{2}\right]}^{2}$$

where we have used the definition of *z* − distribution outlined in the theoretical Section 6 [see Equation (7)]. In Equation (1) τ_1_ = 2.72 × 10^−5^ s is a fixed parameter corresponding to the monodisperse NCs size *d*_1_ = 13.3 nm determined from the data analysis prior to aggregation (see Table 1, sample LHP_A). The other parameters, namely the baseline B, the amplitude *a*_1_ of the NCs, the amplitude *a*_2_ of the aggregates, the *z* − average decay time 〈τ_2_〉_*z*_ and standard deviation $\sigma_{\tau_{2}}$ of the *z* − LogNormal distribution of the aggregates are left as free parameters. Since the decay times scale linearly with the hydrodynamic diameters [see Equation (4)], the diameter distribution of the aggregates mirrors the decay times distribution, i.e., a LogNormal with 〈*d*_2_〉_*z*_ = α 〈τ_2_〉_*z*_ and $\sigma_{d_{2}}=\alpha \;\sigma_{\tau_{2}}$ (being $\alpha =\frac{k_{B}T}{3\pi \eta }q^{2}$). Finally, the amplitudes *a*_1_ and *a*_2_ are related to the *z* −*weighted* relative concentrations of the corresponding species by (*c_i_*)_z_ = *a_i_*/(*a*_1_ + *a*_2_), (*i* = 1,  2), whereas the observed amplitude is given by β_obs_ = (*a*_1_ + *a*_2_)^2^. Noticeably, as shown in Figure 4b,c, the matching between the data and the fitting (solid lines) is quite satisfactory over the entire time‐range of the measurement.

**Figure 5:**
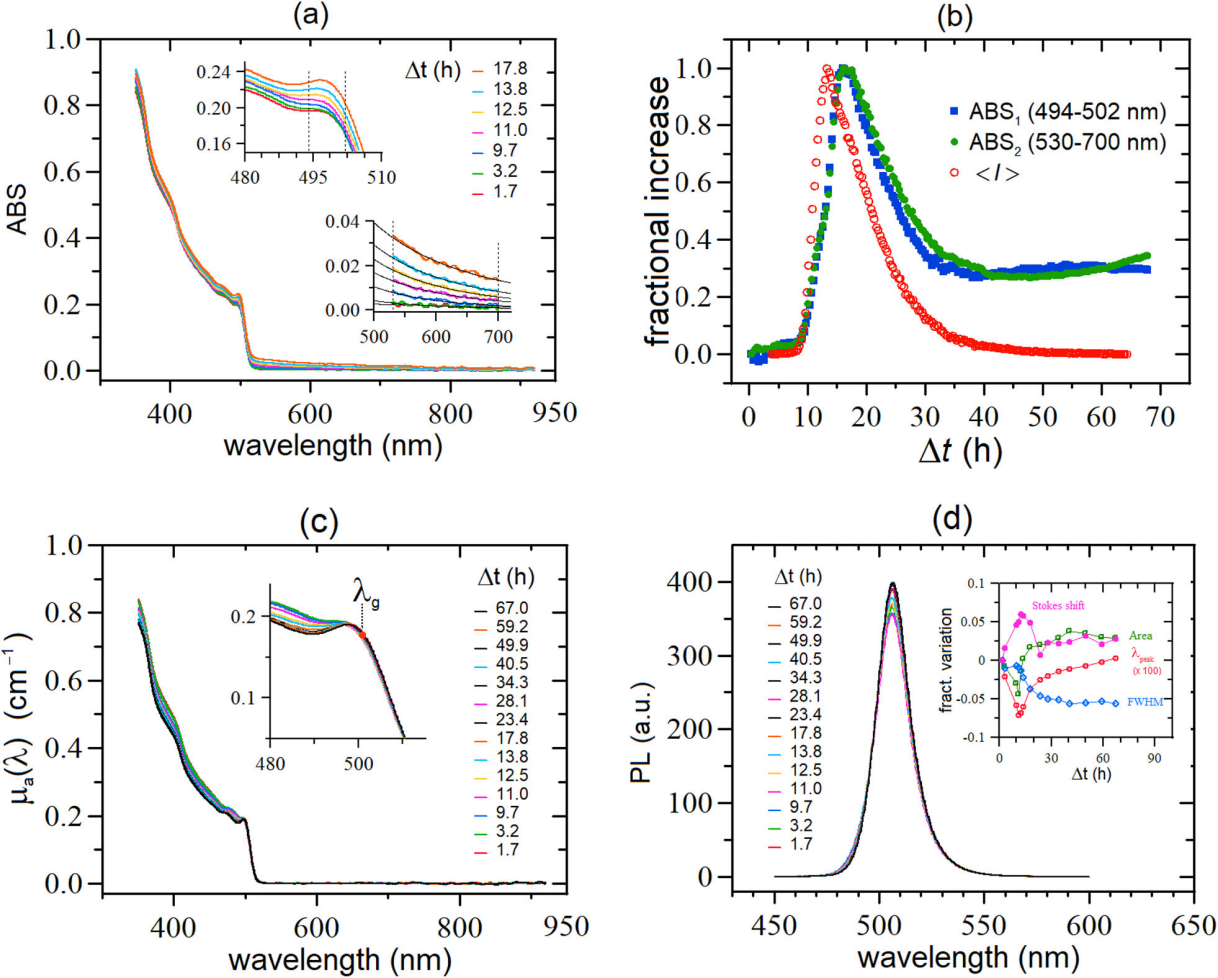
Time behavior of the spectroscopic data taken on sample LHP‐A of Table 1 (same aggregating suspension of Figure 4). a): ABS data for time delays up to Δ*t*  ≈ 18 h; later on, the ABS data stop to increase and, for the sake of clarity, we have not reported this part of the kinetics; the two insets highlight the regions where the relative ABS increase is maximum, i.e., in the 494 − 502 nm and 530 − 700 nm ranges. b): comparison between the time behavior of the fractional increase of the average ABS signals in the two ranges highlighted in the two insets of a) (blue squares and green dots, respectively) with the rescaled DLS data of Figure 4a (red circles). c): absorption coefficients µ_*a*_(λ) for the entire kinetics, up to Δ*t*  ≈ 67 h; the µ_*a*_(λ) curves were extracted from the ABS data by means of the numerical procedure described in the text. The red circle indicates the wavelength λ_*g*_ corresponding to the band gap *E_g_* of the first excitonic transition; the inset shows a magnified plot in correspondence of the absorption peak at ≈ 500 nm. d): the PL spectra exhibit no significative change over the entire kinetics; the inset shows that the relative variations of the PL parameters with respect to their initial values are in the few % range.

Figure 4d shows the behavior of the fitting parameters associated to the distribution of the aggregates as a function of time after dilution, namely: the average diameter 〈*d*_2_〉_*z*_ (red circles) and standard deviation $\sigma_{d_{2}}$ (green squares) of the aggregates. For comparison, we report also the (fixed) diameter *d*_1_ associated to the monodisperse NCs (blue circles) and the (*z* − weighted) average diameter 〈*d*〉_z_ of the *overall* population (NCs and aggregates), with 〈*d*〉_z_ = (*a*_1_ *d*_1_ + *a*_2_〈*d*_2_〉_*z*_)/(*a*_1_ + *a*_2_) (black curve). In parallel, Figure 4e displays the *z* ‐ weighted relative concentration ${\left(c_{2}\right)}_{z}$ of the aggregates (red) while the inset reports also the weight and number concentrations *w* (blue) and *n* −  weighted (green), respectively.

Among the various concentration curves, the most informative one is (*c*_2_)_w_. This curve not only corresponds to the mass‐fraction of the aggregates in suspension, but also equals the number‐fraction of the aggregated NCs, i.e., the number fraction of the NCs that are assembled in the aggregates. Indeed, ${\left(c_{2}\right)}_{w}=\frac{M_{a}}{M_{a}+M_{s}}=\frac{n_{a}}{n_{a}+n_{s}}$ where *M_a_* and *M_s_* indicate the total masses of aggregates and monomeric NCs, whereas *n_a_* and *n_s_* denote the total numbers of aggregated and monomeric NCs, respectively.

As one can notice, before the onset of aggregation (Δ*t* ≤ Δ*t_lat_*), the aggregates concentration (*c*_2_)_z_ in Figure 4e is rather low and difficult to determine accurately from the fit, making the estimates of 〈*d*_2_〉_*z*_ and $\sigma_{d_{2}}$ (Figure 4d) rather uncertain. Later, for Δ*t* ≥ Δ*t_lat_*, ${\left(c_{2}\right)}_{z}$ progressively increases, reaching a value close to unity at $\Delta t\approx \Delta t_{\mathrm{peak}}$. Correspondingly, the determination of the LogNormal distribution parameters becomes progressively more reliable, showing that, at Δ*t* = 13 h, 〈*d*_2_〉_*z*_ = 190 ± 10 nm, $\frac{\sigma_{d_{2}}}{{\left\langle d_{2}\right\rangle }_{z}}\approx 0.35$ and ${\left\langle d\right\rangle }_{z}\approx {\left\langle d_{2}\right\rangle }_{z}$. This trend keeps going on until $\Delta t\approx \Delta t_{\mathrm{peak}}$. At this time, ${\left(c_{2}\right)}_{z}$ starts to decrease due to sedimentation and, as already reported in Figure 4c, the aggregates undergo a second aggregation step that makes them grow up to µm‐size scale, with increasingly larger polydispersity (${\left\langle d_{2}\right\rangle }_{z}\approx 1500\;\mathrm{nm}$, $\frac{\sigma_{d_{2}}}{{\left\langle d_{2}\right\rangle }_{z}}\approx 0.6\;-\;0.7$ at Δ*t* = 40 h). At the end of the sedimentation process, when 〈*I*〉 has returned to its initial value (see Figure 4a), the solution still contains a small fraction of very large aggregates, as shown by the single rightmost points of Figure 4d,e. Finally, we point out that, during the entire aggregation process, the mass‐ and number‐concentrations of the aggregates remain remarkably low (see inset of Figure 4e), indicating that the dominant species present in suspension during the whole kinetics are *always* the individual NC monomers.

Finally, we would like to highlight that, due to the intrinsic stochastic nature of the aggregation process, the intriguing kinetics of the NCs’ aggregation described in Figure 4 would not have been revealed using separate instruments. Analogous situations are also described in literature, as shown in ref. [44] As a further check of the correct interpretation of the complex analysis of the DLS data, we cross validated our results via SLS measurements, taken on the same sample of Figure 4, by using a homemade instrument available at our laboratory. Details are reported in Section S8 (Supporting Information).

#### 4.1.2 ABS Data

The effects of the aggregation kinetics of the ABS data are displayed in **Figure** **5a**, where for clarity, we have reported only the growing part of the kinetics; here the effects of aggregation are less evident than those in observed in Figure 4a, but still clearly evident, mostly at wavelengths where the pristine sample is transparent (λ ≥ 550 nm, bottom right inset) and in correspondence of the first absorption peak (λ≈500nm, upper left inset). In particular, the absorbance increase at λ ≥ 550 nm is entirely due to light scattering from the aggregates that grow in size until to Δt≈18h when they reach sizes 〈*d*_2_〉  ≥ 500 nm (as estimated from the DLS analysis). Later, while they still keep growing, they start to settle down (as shown in Figure 4b); as a consequence, the ABS signal first attains a plateau and then starts to slowly decrease.

The full time‐dependent behavior can be appreciated in Figure 5b where we compare the (normalized) fractional increase of the average ABS in correspondence of the two regions highlighted in the two insets of Figure 5a [i.e., 494−502nm (blue curve) and 530−700nm (green curve)] with the DLS‐derived trace (red curve). All the three curves exhibit similar behavior: after the same latency time, they start to increase, attaining a maximum at similar Δ*t* values and, later, slowly decrease to a plateau that, in the case of DLS, equals its initial value, whereas, for ABS, is ≈30% of the peak amplitude. Thus, also the ABS data are consistent with the formation of large NCs aggregates that grow in time, increasingly scatter more light, and ultimately precipitate off the suspension in about two days. The fact that the plateau amplitudes of the two ABS data do not relax back to their initial values (as it occurs for the DLS data) is attributed to the presence of residual large aggregates ($d_{\mathrm{agg}}\approx 1.5\;\mu m$, see Section 4.2.1), still suspended in solution, as qualitatively confirmed by the non‐single exponential decay of the final *g*_2_(τ) curves reported in Figure 4c. Differently, the scattering of these large aggregates does not give any contribution to the DLS signal detected at θ = 90°, which therefore relaxes to its initial value. For this same reason (that is, large aggregates do not contribute to the scattered intensity at θ = 90° because they mainly scatter light in the forward direction), the peak of the DLS curve slightly anticipates those exhibited by the two ABS curves.

We can combine the results of Figure 5b with those of Figure 4e for extracting information on the amount of NCs that have precipitated off the suspension during the sedimentation process. To this aim, let us define ${\left(c_{2}^{*}\right)}_{w}=\frac{n_{a}}{n_{0}}$ as the number‐fraction of aggregated NCs that are still in suspension relative to the initial number of NCs *n*_0_ (*n*_0_ is clearly a constant). If we suppose that sedimentation starts only after the peak position has been attained (Δt≈13h), at that time $(c_{2}^{*})w=(c_{2})w\;\approx \;0.1$. We know also from Figure 5b that not all these aggregates precipitate off the suspension because ABS does not relax to zero, but stabilizes at about ≈30% of its peak. Therefore, about ≈30% of all the aggregated NCs remains in suspension, implying that their final fraction with respect to the initial number of NCs is ${\left(c_{2}^{*}\right)}_{w}\left(t\to \infty \right)\;∼\;0.1\;\times 0.3=3\%$. Correspondingly, ≈7% of the NCs have precipitated off the suspension in aggregated form.

Additionally, we point out that we can extract the *true* absorption spectrum $\mu_{a}\left(\lambda \right)$ from the ABS data shown in Figure 5a by exploiting the fact that in the 530 − 700 nm region there is no absorption and the ABS signal in this region is *entirely* due to scattering. Thus, we fitted the ABS data in this range with a power law function (which is the expected functional dependence for sub‐micron aggregates),^[^ ^45^, ^46^ ^]^ we extrapolated the fitted values to the region where is absorption is present, and we finally subtracted such a contribution from the overall ABS data. This analysis is shown in Figure 5c where one can notice that, near the rising shoulder, all the $\mu_{a}\left(\lambda \right)$ traces collapse on a single master curve that is *independent* of aggregation. The only feature of the $\mu_{a}\left(\lambda \right)$ spectrum that exhibits a significant change in time is the increase of the peak contrast, clearly visible in the figure inset.

As a consequence of this analysis, we found that the energy gap of the first excitonic transition *E_g_* [and corresponding wavelength λ_*g*_ = *hc*/*E_g_*, where *h* is the Plank constant and *c* the speed of light, with standard units λ_*g*_(nm) = 1239.8/*E_g_* (eV)] is constant *over the entire process*. The accurate estimate of λ_*g*_ (or *E_g_*) was carried out by determining the minimum of the second derivative of the $\mu_{a}\left(\lambda \right)$ spectrum, $\left[d^{2}\mu_{a}\left(\lambda \right)/{\left(d\lambda \right)}^{2}\right]$,^[^ ^47^, ^48^ ^]^ or alternatively, the minimum of $d^{2}\mu_{a}\left(E\right)/{dE}^{2}$ (*E* = *hc*/λ). For the entire set of data shown in Figure 5c, we obtained $\lambda_{g}=501.4\pm 0.1\;\mathrm{nm}$ and *E_g_* = 2.473 ± 0.001 eV. The point corresponding to λ_*g*_ is shown in red in the inset of Figure 5c.

Finally, as already pointed out at the end of Section 3, an accurate estimate of *E_g_* does not guarantee a reliable estimate of the NC edge length $\ell_{\mathrm{ABS}}$, largely depending on the choice of the calibration curve. However, our analysis shows quite clearly that, since *E_g_* does not change during this period, the size of the individual NCs remains unchanged, regardless of which curve $\ell_{\mathrm{ABS}}\left(E_{g}\right)$ is adopted.

#### 4.1.3 PL Data

As PL kinetics are concerned (Figure 5d), the effects of aggregation are rather negligible, with only a slight increase of the peak amplitude, without almost any change in the spectral shape. By analyzing all the curves of Figure 5d, we recovered average and variance values of the peak parameters distribution: $\lambda_{peak}=507.4\pm 0.1\;\mathrm{nm}$ and FWHM=18.5±0.2nm and (by using the data of Figure 5a) the Stokes shift ΔEs=6.0±0.1nm. In energy units they correspond to $E_{peak}=2.443\pm 0.001\;\mathrm{eV}$, FWHM=89.0±1meV and ΔEs=29±1meV.

The remarkable stability of all the PL curves is highlighted in the inset of Figure 5d, where the fractional variations of the various parameters (λ_*peak*_, Area, FWHM, Stokes shift) with respect to their initial values are reported as a function of time. As one can observe all parameters vary less than a few percent, with the peak position being by far the most stable one, with a relative variation lower than 7 × 10^−4^. This apparent absence of specific features can be indeed turned into a highly informative point: consistently with the interpretation of the DLS data, the aggregation occurs without NCs coalescence, being the result of an incoherent (i.e., randomly‐ oriented) assembly of NCs, which can reach micrometer sizes.

### 4.2 Aggregation Kinetics of OAc/OAm Stabilized CsPbBr_3_ Perovskites NCs upon 1:100 Dilution: Long‐Times Behavior (3–75 days)

To investigate the long‐term stability and the kinetic behavior of the OAc/OAm stabilized samples reported in Table 1, we periodically (re)measured them for a prolonged time, lasting ≈2–3 months). The DLS and PL and measurements were carried out by using the same experimental setting described in Section 3. During this entire period, except for the measurement sessions, all samples were kept at room temperature, in the dark, without agitation and sealed in their own cuvettes. Each measurement was repeated twice, first with the sample at rest and then, immediately later, after gentle shaking. The time interval between measurements pairs was of several days, thus allowing for an efficient sedimentation of the NCs aggregates after shaking. In the following we describe in some detail the results related to the same sample characterized in Figure 4 and Figure 5, *i.e*., sample LHP‐A of Table 1, obtained under a 1:100 dilution.

#### 4.2.1 DLS Data

**Figure 6:**
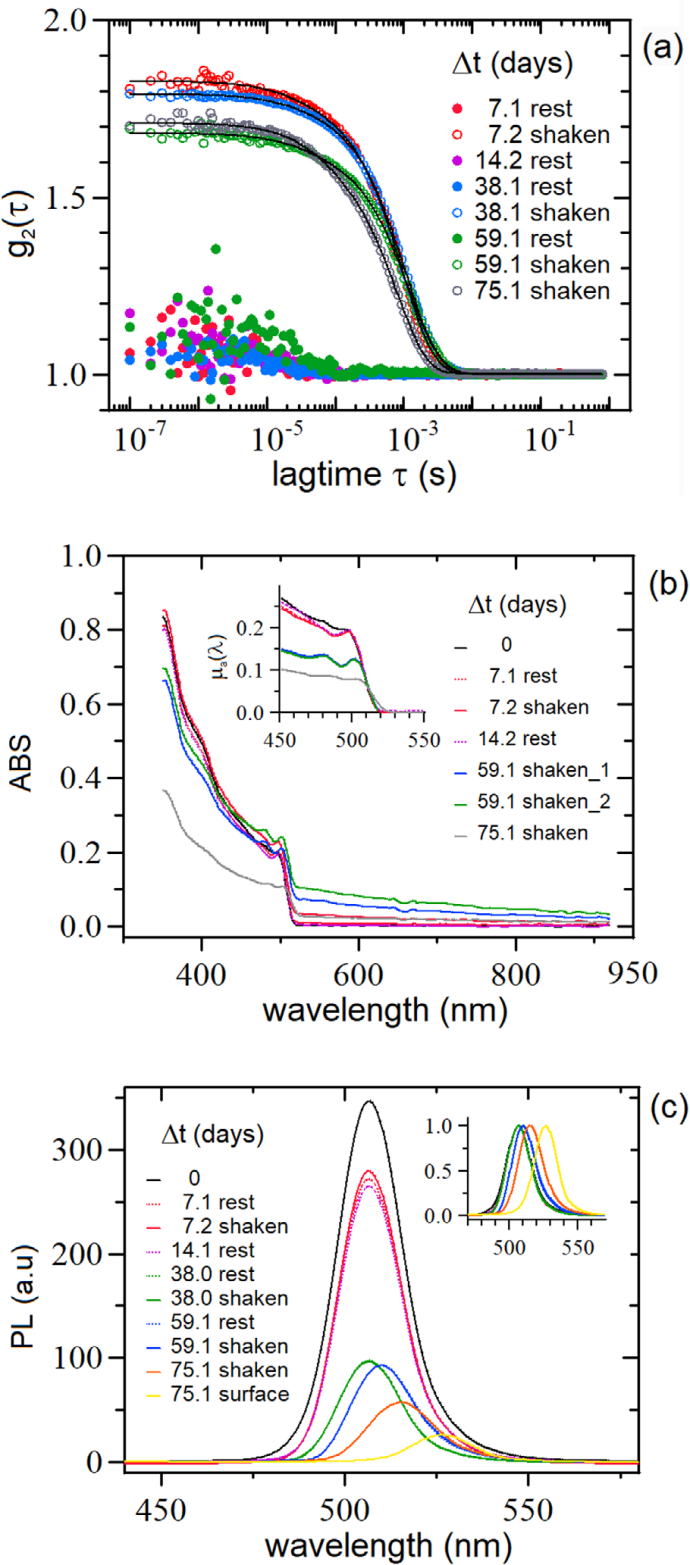
a) DLS, b) ABS and c) PL data for the sample LHP‐A of Table 1 discussed in Figure 4 and Figure 5, taken during a prolonged time interval (7–75 days). In the inset of (b), the ABS data are shown after scattering subtraction. In (c), the last yellow curve was taken from the NCs stuck on the surface of the empty cuvette (see text); the inset shows PL data normalized to their maxima.

**Figure** **6**, panel (a) reports an example of the DLS data taken over a period of time ranging between 3 and 75 days. Data corresponding to the samples at rest were not fitted due to excessive noise. As expected, after shaking the cuvette (with the aggregates brought back in suspension), the average intensity 〈*I*〉 rises to levels similar to 〈*I*〉_*peak*_ values of Figure 4a (data not shown) and, correspondingly, the *g*_2_ curves become much smoother with β_obs_ values and decay times larger than the ones measured with the samples at rest. These data were fitted by using Equation (1), where in this case, beyond 〈*d*_2_〉_*z*_ and $\sigma_{d_{2}}$, also τ_1_ was left as a free parameter. From the resulting values, we observed a slight tendency toward progressively increasing the average NCs diameter *d*_1_, ranging from 12.3±0.7nm at Δt≈21days to 30±2nm at Δ*t* = 75.1 days. The corresponding edge lengths vary [by use of Equation (5)] from $L_{1}=9.3\pm 0.5\;\mathrm{nm}$ to $L_{1}=22.7\pm 0.5\;\mathrm{nm}$, suggesting the possibility that some of the original NCs coalesce into larger coherent crystalline domains.^[^ ^41^ ^]^

The other two parameters of interest, 〈*d*_2_〉_*z*_ and $\sigma_{d_{2}}$, differ only slightly from those found in the first regime (with Δ*t* ≥ 40 h, shown in Figure 4d, i.e., with 〈*d*_2_〉 falling in the µm (or larger) range and $\frac{\sigma_{d_{2}}}{{\left\langle d_{2}\right\rangle }_{z}}\;\approx \;0.5$). In this case, since we expect that the aggregates do not retain the cubic shape of the individual NCs, no morphological shape correction was applied. As to the concentrations, the situation closely resembles the one described in Figure 4e, with the presence in solution of a remarkably small fraction of large micro‐sized (and highly polydisperse) aggregates and a large number of remnant monomeric NCs.

#### 4.2.2 ABS Data

Analogously to DLS data of Figure 6a, the whole set of ABS spectra is reported in Figure 6b. As expected, the ABS data of the samples at rest are almost null for wavelengths where there is no absorption (λ ≥ 550 nm), whereas, after shaking, long tails due to scattering of the NC aggregates are observed. Overall, the visual superimposition of all curves in the region corresponding to the first ABS peak (λ≈500 nm) is rather chaotic and difficult to interpret. However, after subtraction of the scattering contribution (as described in Section 4.1.2), the behavior of µ_*a*_(λ) curves (portrayed in the inset of Figure 6b) becomes quite clear, showing that, as time goes by, the first peak becomes progressively more structured without any change of its amplitude, which tends to dampen out only at very long times (Δ*t*  ≈  60 days). Note that the two curves taken at the same time Δ*t*  ≈ 59.1 days (blue and green) show differences due to different degrees of shaking (that correspond to different extents of scattering), but once corrected, they nicely superimpose one each other, as expected.

By carrying out the second derivative analysis, we observed that λ_*g*_ increased from 501.4 nm at Δ*t* ≈ 3 days to 507.7 nm at Δ*t* ≈ 75 days. Correspondingly, *E_g_* decreased from 2.473 to 2.442 eV with estimated edge lengths increasing from $\ell_{\mathrm{ABS}}=7.4$ to $\ell_{\mathrm{ABS}}=8.8\;\mathrm{nm}$, as recovered from the calibration curve.^[^ ^36^ ^]^ While the absolute values of $\ell_{\mathrm{ABS}}$ are hard to rely on (due to their dependence on the sizing curve utilized), we noticed that their relative increase $\Delta \ell_{\mathrm{ABS}}\left(75\right)/\ell_{\mathrm{ABS}}\left(3\right)\;\approx 20\%$ appears to be curve‐independent, thus supporting the hypothesis of NCs coalescence. A similar behavior was observed on all the samples listed in Table 1 (at all dilutions), as shown in Figure S4 (Supporting Information).

#### 4.2.3 PL Data

PL data are shown in Figure 6c. With respect to the initial (Δt≈3days) PL spectrum, the main features of the following spectra are: (i) a remarkable reduction of the PL intensities down to ≈80% of their initial values and, (ii) a red‐shift of the PL peak maxima, which increases from the initial value of λ_*peak*_ ≈  507.4 nm to λ_*peak*_ ≈ 516 nm at Δ*t*  ≈  30 − 40 days. As for the DLS and ABS data, the increase of λ_*peak*_ is a further support to the hypothesis of NCs coalescence and was confirmed for all the samples listed in Table 1 (at all dilutions), as shown in Figure S5 (Supporting Information).

No appreciable difference between the PLs of “at rest” and shaken samples is observed, implying that PL is not affected by the process of incoherent NCs aggregation.

The bottom trace of Figure 6c (yellow, taken at Δ*t* ≈ 75 days) shows the largest red shift and refers to PL data taken from the surface of the cuvette once the suspension has been removed. Here, NCs stuck to the surface formed a rather uniform layer, clearly visible by the eye. While describing and interpreting this phenomenon (which was observed in all examined samples) falls beyond the focus of this study, we point out that the presence of this layer does not introduce any self‐absorption effect in our PL and ABS measurements because we checked for all samples that PL and ABS spectra measured in the original cuvettes (with the layer present) are identical to the ones measured after the suspensions were transferred into clean ones (data not shown).

## 5 Discussion and Conclusion

In this work, we have developed a simple instrument that utilizes commercial components to perform simultaneous Dynamic Light Scattering (DLS), Absorbance (ABS), and Photoluminescence (PL) measurements. To the best of our knowledge, this is the first time that such an instrument is proposed in the scientific literature. It offers two key advantages compared to the traditional laboratory approach, which works by using distinct and separate instruments: firstly, it allows for the simultaneous analysis of the very same sample, overcoming limitations arising from measurements being performed on distinct specimens, under varying experimental conditions, at different times; secondly, it enables time‐resolved monitoring of the kinetic evolution of NCs' colloidal stability or instability. Designed for standard laboratory use, the instrument provides a quick analysis within ≈100%s, a duration significantly shorter than typical timescales involved in aggregation kinetics or other colloidal instability‐related phenomena. Furthermore, since it is well known^[^ ^18^ ^]^ that DLS and ABS probe different length scales and static‐dynamical properties, the combined use of simultaneous spectroscopic and light scattering techniques is expected to be much more powerful than using the single techniques independently,^[^ ^18^ ^]^ as shown in various examples taken from the literature.^[^ ^45^, ^46^, ^49^ ^]^

All these features make the instrument highly attractive for the physical‐chemical characterization of colloidal suspensions that, representing a paradigmatic example for many soft materials, are of great interest for both basic research and technological applications.^[^ ^50^, ^51^ ^]^ In the context of this work, we demonstrated the suitability of the instrument for characterizing time‐resolved processes through a case study on the long‐term (in) stability of OAc/OAm‐capped colloidal CsPbBr_3_ NCs suspensions upon dilution. After dilution, the gradual desorption of ligands from the surface results in a reduced steric shielding between NCs. Consequently, the suspension may become unstable, leading to the formation of either incoherent (i.e., randomly‐arranged) aggregates, or to the coalesce into larger crystalline domains.

Our findings show that, in the case of a suspension with an original concentration of $\approx 7\mathrm{mg}\;{\mathrm{mL}}^{-1}$ diluted by a factor 1:100, after a latency time of $\Delta t_{\mathrm{lat}}\;\approx \;8\;h$, the NCs begin to progressively scatter more light, a phenomenon that was attributed to the formation of increasingly larger aggregates that can reach micro‐sized dimensions (estimated by DLS). At the same time, while still growing, the aggregates start to settle down and eventually (within about two days) disappear from the suspension. During this time, the spectroscopic features (absorption coefficient µ_*a*_(λ) and PL spectrum) of the suspension remain unchanged, suggesting that aggregation occurs without any coalescence, being the result of an incoherent (i.e., randomly oriented) NCs assembly. Later, for a period of three months, the suspension returned to be quite stable, without any further formation of aggregates and with only a marginal change of its spectroscopic features that we attributed to partial coalescence of the original NCs.

A key limitation of the instrument proposed in this work is its relatively low sensitivity when DLS is performed on very small (fluorescent) NCs samples (typically *d* ≤ 5 − 10 nm) as the correlation function becomes rather noisy. In that case, employing a more powerful laser source does not help in enhancing the S/N ratio of the data, and the sole remedy is to collect the DLS signal for a very long measuring time or, alternatively, to average many correlation functions. The result is a significant decrease in the instrument time resolution, which may jeopardize its ability to analyze the time evolution of the NC samples. A possible way out for this issue is to use an IR laser that does not excite any fluorescence, as done in some commercial DLS instruments (see for example the DynoPro ZetaStar, Wyatt Technology, operated at 785 nm).

Finally, we would like to point out that, while the current status of our instrument allows for simultaneous DLS/ABS/PL measurements, its further development with the implementation of additional spectroscopic techniques seems to be feasible. For example, employing a pulsed laser would enable the observation of the sample *time‐resolved* PL spectrum, while utilizing a white laser would allow for the measurements of the *PhotoLuminescence Emission* (PLE) spectra. A bit more complex would be the integration of the instrument with a SAXS line, which offers a precise and sensitive assessment of the NC size, shape and may reveal the fractal morphology (if any) of the aggregates. The last idea is rather appealing but not so straightforward, though a few examples where DLS and SAXS have been combined in the same *laboratory* setup have appeared.^[^ ^52^, ^53^ ^]^

## 6 Theoretical Section

In this section we recall the fundamental principles and the basic equations that are behind the techniques we used in this work: dynamic light scattering, absorbance and photoluminescence.

### 6.1 Dynamic Light Scattering

Dynamic Light Scattering (DLS) is based on the determination of the translational diffusion coefficient *D* of particles freely moving in a fluid. The method works by measuring the normalized auto‐correlation function

(2) $$g_{2}\left(q,\tau \right)=\left\langle I\left(q,t\right)I\left(q,t+\tau \right)\right\rangle /{\left\langle I\left(q\right)\right\rangle }^{2}$$

of the Intensity *I*(*q*, *t*) scattered by the sample at a given wavevector ***q*** with modulus q=(4π/λ)nsin(θ/2), where θ is the scattering angle, λ the vacuum laser wavelength, and *n* the refraction index of the solvent. For what it matters here, the DLS method works also with fluorescent particles,^[^ ^54^ ^]^ i.e., with particles that scatter the laser light and, at the same time, emit fluorescent light that acts as a (undesired) background signal, which is completely equivalent to other background (i.e., not sample‐related) signals, such as the detector dark count. The two (scattering and background) contributions sum up incoherently so that the total detected intensity is *I*_tot_ = *I*_sca_ + *I*_bkg_. In the case of an ideal, monodisperse sample (e.g., made of scattering and fluorescing particles of identical size) measured under ideal scattering conditions (e.g., dispersed in a dilute suspension with no interactions among particles and no multiple scattering), the behavior of *g*_2_ as a function of the lag‐time τ and the *q* vector reads:

(3) $$g_{2}\left(q,\tau \right)=B+\beta_{\mathrm{obs}}{\left[\exp\left(-\tau /\tau_{c}\left(q\right)\right)\right]}^{2}$$

where τ_*c*_(*q*) is the (field) decay time, *B* is the asymptotic behavior of *g*_2_ for τ→∞ (ideally *B* = 1). The term $\beta_{\mathrm{obs}}=\beta_{\mathrm{ca}}\frac{{\left\langle I_{\mathrm{sca}}\right\rangle }^{2}}{{\left\langle I_{\mathrm{tot}}\right\rangle }^{2}}$ is a dimensionless factor (0 ≤ β_obs_ ≤ 1)^[^ ^55^ ^]^ that depends on the relative strength between the scattered and background intensities and on the spatial coherence factor β_ca_, related the number *N_ca_* of coherence areas detected by the collection optics. When *N_ca_*  ≫  1, $\beta_{\mathrm{ca}}∼\frac{1}{N_{ca}}$.

By fitting the DLS data to Equation (3) (with *B*, β_obs_ and τ_*c*_ as free parameters) one can recover the translational diffusion coefficient *D* = (τ_*c*_ *q* ^2^)^−1^ and, in turn, by using the Stokes‐Einstein relation,^[^ ^56^ ^]^ the hydrodynamic diameter:

(4) $$d_{h}=\frac{k_{B}T}{3\pi \eta D}=\frac{k_{B}T}{3\pi \eta }q^{2}\tau_{c}$$

where *k*_B_ is the Boltzmann constant, *T* the absolute temperature and η the viscosity of the fluid. Whereas for a spherical particle *d_h_* corresponds to its geometrical diameter independently of the particle internal structure, for other shapes the correspondence between *d_h_* and the particle geometrical features is not straightforward. Given the relevance to the perovskite NCs discussed in this work, we consider here only the case of cubic‐like particles. For an ideal cubic particle with a (sharp) edge length *L*, such a correspondence is:

(5) $$d_{h}=1.32\;L$$

where the factor 1.32 was numerically derived.^[^ ^57^, ^58^ ^]^ Note that 1.32 is a value slightly larger (by ≈6%) than ${\left(6/\pi \right)}^{1/3}\approx 1.24$, which is the factor relating the diameter of a sphere to the edge length of a cube with the same volume.^[^ ^58^ ^]^ Equation 5 can be generalized to cubic‐like particles characterized by a smoothly changing shape (from a cube to a sphere) depending on a single morphological parameter α. For such a particle the hydrodynamic diameter reads:

(6) $$d_{h}=\alpha \;{\left(6/\pi \right)}^{1/3}\;V^{1/3}$$

where *V* is the particle volume. As shown in Figure 2 of Ref. [58], for perfect cubes of edge length *L*, α = 1.06, and, since *V* = *L* ^3^, the same result of Equation (5) is recovered. As the particle shape becomes more and more spherical‐like (for example by beveling or rounding its edges), the parameter α monotonically decreases approaching the asymptotic value α = 1, Thus, for a sphere of geometrical diameter *d*, since *V* = (π/6)*d* ^3^, we get the expected result *d_h_* = *d*. The applicability of Equation (6) requires the exact knowledge of the particle shape, an information that might be difficult to obtain as in the case of our NCs due to the flexible and partially permeable nature of the capping ligands. Hence, in the rest of this work we will use Equation (5), fully aware of the fact that it represents an upper bound to the relation between *d_h_* and *L*.

Finally, we discuss how the DLS technique can be employed to characterize polydisperse samples. If *P_z_* (τ_*c*_) indicates the (normalized) intensity‐weighted distribution function of decay times, Equation (3) becomes:^[^ ^18^ ^]^

(7) $$\;g_{2}\left(q,\tau \right)=B+\beta_{\mathrm{obs}}{\left[\int_{0}^{\infty }P_{z}\left(\tau_{c}\right)\exp\left(-\frac{\tau }{\tau_{c}}\right)d\tau_{c}\;\right]}^{2}$$

Note that *P_z_* (τ_*c*_) is an *intensity* distribution of decay times, where the contribution of particles with a given decay time τ_*c*_ is weighted by their time‐averaged scattered intensity 〈*I*(*q*)〉. In the case of small particles with diameters *d* ≪ λ, such an intensity is independent of *q* and scales as $\left\langle I\right\rangle \;∼\;m^{2}\;∼\;d^{6}$. Thus, under this assumption, intensity distributions are equivalent to *m* ^2^ −  distributions, which are much more skewed toward large particles than weight (w) or number (n) distributions. Quantitatively, $P_{z}\left(\tau_{c}\right)\;∼\;d^{3}P_{w}\left(\tau_{c}\right)\;∼\;d^{6}P_{n}\left(\tau_{c}\right)$.

### 6.2 Absorbance

The absorbance (ABS) measurement of a NCs colloidal dispersion is based on the Lambert‐Beer law that relates the power *P*_0_(λ) incident on the sample to the transmitted power *P_T_* (λ) by:

(8) $$P_{T}\left(\lambda \right)=P_{0}\left(\lambda \right)\;e^{-\mu_{e}\left(\lambda \right)L}$$

where λ is the vacuum wavelength of the incident radiation, *L* the sample optical path, and *µ*_*e*_(λ) = *µ*_*a*_(λ) + *µ*_*s*_(λ) is the λ‐dependent *extinction* coefficient given by the sum of the absorption (*µ*_*a*_) and scattering (*µ*_*s*_) coefficients. These coefficients scale linearly with the sample concentrations as *µ*_*a*_(λ) = σ_*a*_ *n* and *µ*_*s*_(λ) = σ_*s*_ *n*, where *n* is the number sample concentration [cm^−3^], whereas σ_*a*_ and σ_*s*_ are the absorption and scattering cross sections [cm^2^], respectively.

Equation (8) is often reported as:

(9) $$P_{T}\left(\lambda \right)=P_{0}\left(\lambda \right)\;10^{-A\left(\lambda \right)}$$

where $A\left(\lambda \right)=\frac{\mu_{e}\left(\lambda \right)L\;}{\ln10}$ is the sample absorbance. It should be pointed out that, although it is common to associate *A*(λ) to the sample absorption spectrum, the quantity that is actually measured in the case of a NCs dispersion is *µ*_*e*_(λ). Thus, accurate measurements of the NCs absorption spectrum can be carried out *only* by correcting *A*(λ) for the scattering contributions, which in the presence of large particles (as in the case of large NCs aggregates) might not be negligible.

The correct determination of *µ*_*a*_ is of basic importance for an accurate estimate of the energy gap *E_g_* associated to the first excitonic transition in NCs. Such parameter can be exploited to estimate, via various empirical calibration curves *L*(*E_g_*) available in literature, the average NCs sizes (edge Length) *L*. A reliable estimate of *E_g_* [or equivalently of the wavelength λ_*g*_ = *hc*/*E_g_*, where *h* is the Plank constant and *c* the speed of light; with standard units λ_*g*_(nm) = 1239.8/*E_g_* (eV)] can be carried out by using standard numerical methods. One commonly adopted approach is based on the multi‐component Gaussian fitting^[^ ^48^ ^]^ of the *µ*_*a*_(λ) spectrum, where λ_*g*_ is taken as the maximum of the average values of all the Gaussians utilized in the fitting process. This method is highly background‐dependent, from which the importance of using *µ*_*a*_(λ) instead of *µ*_*e*_(λ). An alternative approach involves finding the minimum of the second derivative the spectrum *µ*_*a*_(λ), i.e. the minimum of $d^{2}\mu_{a}\left(\lambda \right)/d\lambda^{2}$ ^[^ ^47^, ^48^ ^]^ or alternatively, the minimum of $d^{2}\mu_{a}\left(E\right)/dE^{2}$. This method is rather immune to any broad and smoothly changing background^[^ ^48^ ^]^ present in the analyzed curve (such as the scattering profiles mentioned above), implying that the derivative analysis carried out on the *µ*_*e*_ or *µ*_*a*_ data would provide almost identical results. Given its simplicity, we adopted this method for deriving λ_*g*_ (or *E_g_*) throughout all this work.

### 6.3 Steady–State Photoluminescence (PL) and Self‐Absorption Correction

**Figure 7:**
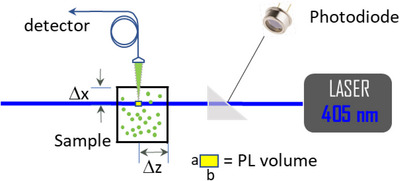
Scheme of PL detection measurement. The use of a square cuvette together with the knowledge of the sample extinction coefficient *µ*_*e*_(λ) and the distances Δ*z* and Δx allow us to correct the PL spectrum for the self‐absorption and scattering effects reported in Equation (10).

The steady–state Photoluminescence (PL) spectrum of a NCs colloidal suspension can be easily measured following the scheme depicted in **Figure** **7**. The sample, contained in a standard square cuvette, is shined with a CW blue laser and the fluorescence intensity coming from the PL volume (yellow square in Figure 7) is detected at 90° by using a collecting lens coupled to a multi‐mode optical fiber. The measured signal $PL^{\mathrm{meas}}\left(\lambda \right)$ is related to the actual sample photoluminescence $\mathrm{PL}(\lambda_{0},\lambda )$ by:

(10) $$PL^{\mathrm{meas}}\left(\lambda \right)=P_{\mathrm{inc}}\left(\lambda_{0}\right)\;\;\mathrm{PL}\left(\lambda_{0},\lambda \right)\;e^{-\mu_{e}\left(\lambda_{0}\right)\Delta z}\;e^{-\mu_{e}\left(\lambda \right)\Delta x}$$

where *P_inc_* (λ_0_) is the incident laser power (measured via the photodiode indicated in the figure), $\mu_{e}\left(\lambda \right)$ is the extinction coefficient of the sample, and the two exponentials are the self‐absorption correction factors. The first one depends on the distance Δ*z* travelled by the laser until reaching the PL volume, whereas the second one depends on the distance Δx travelled by the PL light until exiting the cuvette. Notice that Equation (10) is valid only when the PL volume can considered point‐like, i.e., when its linear dimensions (*a*,  *b*) are much smaller than the distances Δ*z* and Δx. When this assumption is not fulfilled, Equation (10) must be integrated over the entire the PL volume, but this is not the case with the detection scheme of Figure 7. Notice also that: i) the first correction term is λ − independent, thus introducing only a scaling factor in the measurement without affecting the shape of $PL^{\mathrm{meas}}\left(\lambda \right)$; ii) in Equation (10) we have used $\mu_{e}\left(\lambda \right)$ and not $\mu_{a}\left(\lambda \right)$ because the solid angle of the collection optics of the PL detector is, in our case, rather small and consequently the fluorescent light that is scattered by the sample does not affect the measurement reentering the collection optics. In the (more common) case where PL measurements are carried out by using an integration sphere (where the fluorescent light is collected over the entire 4π steradiants solid angle), $\mu_{a}\left(\lambda \right)$ must be used instead of $\mu_{e}\left(\lambda \right)$ and a formula different from Equation (10) must be used.

Finally, we mention that the energy $E_{PL_{max}}$ corresponding to the maximum of the photoluminescence band can be used to compute the so‐called Stokes shift $\Delta Es=E_{g}-E_{PL_{max}}$ where *E_g_* is the energy of the first electronic transition (see previous section). As for *E_g_*, Δ*E* can be related to the average NCs sizes (edge Length) *L* via other empirical calibration curves *L*(Δ*E*) available in literature,^[^ ^37^, ^59^ ^]^ but this method is less reliable than the one based on the energy gap *E_g_*, and it has not used in this article.

## Conflict of Interest

The authors declare no conflict of interest.

## Supporting information



Supporting Information

## Data Availability

The data that support the findings of this study are available from the corresponding author upon reasonable request.
