# Supplementary material for: Simultaneous Dynamic Light Scattering, Absorbance and Photoluminescence Measurements of Colloidal Nanoparticles. Application to Colloidal Stability and Aggregation Kinetics of CsPbBr3 Nanocrystals
Source: Small Methods. 2025 Oct 30;9(11):e00304. doi: 10.1002/smtd.202500304 (PMC12641377; doi:10.1002/smtd.202500304)
Supplement: Supplementary file 1 — Supporting Information [file SMTD-9-e00304-s001.pdf]

SUPPLEMENTARY INFORMATION

Simultaneous dynamic light scattering, absorbance and photoluminescence measurements of colloidal nanoparticles. Application to colloidal stability and kinetics of aggregation of $CsPbBr_3$ nanocrystals.

Pietro Anzini[1,2]*, M. Chiara Bossuto[1], Mara Colombo[1], Anna Vivani[1], Ihor Cherniukh[3,4], Maryna I. Bodnarchuk[3,4], Maksym V. Kovalenko[3,4], Federica Bertolotti[1], Antonietta Guagliardi[5], Norberto Masciocchi[1] and Fabio Ferri[1,2]*

[1]Dipartimento di Scienza e Alta Tecnologia and To.Sca.Lab, Università degli Studi dell'Insubria, Via Valleggio 11, I-22100 Como, Italy

[2]CLIP-Como Lake Institute of Photonics, via Valleggio 11, Como I-22100, Italy

[3]Department of Chemistry and Applied Bioscience, Institute of Inorganic Chemistry, ETH Zurich, Vladimir Prelog Weg 1, CH-8093 Zurich, Switzerland

[4]Laboratory for Thin Films and Photovoltaics, Empa—Swiss Federal Laboratories for Materials, Science and Technology, CH-8600 Dubendorf, Switzerland

[5]Istituto di Cristallografia (IC) and To.Sca.Lab, Consiglio Nazionale delle Ricerche (CNR), via Valleggio 11, Como, I-22100, Italy

Index:

1 – Sample preparation

2 – Analysis of the TEM images

3 – Sizing curves from ABS measurements

4 – Aggregation kinetics of all the OAc/OAm stabilized $CsPbBr_3$ perovskite NCs 4

5 – Estimate of the relative scattering and fluorescence signals

6 – Comparison between DLS data taken with green and red lasers

7 – Comparison between DLS-ABS-PL results taken with our setup and commercial instruments.

8 – Characterization of $CsPbBr_3$ perovskite NCs aggregation by Static Light Scattering

9 – Estimation of noise associated to DLS data and corresponding detection limits.

10 – Top view photo of the actual instrumental setup

1 – Sample preparation

$CsPbBr_3$ nanocrystals (NCs) **LHP-A** were synthesized following the method reported in Ref.[1] with some modifications. In a 25 ml three-neck flask, $PbBr_2$ (110 mg, ABCR, 98%) was degassed three times, suspended in 1-octadecene (10 ml, ODE, Sigma-Aldrich, 90%, distilled), and degassed three times again at room temperature. The suspension was then rapidly heated to 190 °C. When the temperature reached 125 °C, oleic acid (1 ml, OAc, Sigma-Aldrich, 90%, vacuum-dried at 100 °C) and oleylamine (1 ml, OAm, Strem, 97%, distilled) were injected. At 190 °C, cesium oleate solution in ODE (0.9 ml, preheated to about 100 °C) was injected. The reaction mixture was immediately cooled to room temperature with an ice bath. The crude solution was centrifuged at 11500 rpm (equivalent to 18190 relative centrifugal force) for 5 min. The supernatant was discarded, and the precipitate was dispersed in toluene (0.6 ml, Sigma-Aldrich, anhydrous, 99.8%). The solution was centrifuged again at 10000 rpm for 3 min, the precipitate was discarded, and the supernatant was collected and diluted with additional toluene (0.5 ml).

Cesium carbonate stock solution was prepared by reacting Cs_2CO_3 (0.2 g, Sigma-Aldrich, 99.9%) with OA (0.6 ml) in ODE (7.5 ml) in a 25 ml flask. First, the mixture was stirred under vacuum for 20 min at 100 °C and then heated under N_2 to 120 °C until all the Cs_2CO_3 dissolved.

$CsPbBr_3$ NCs **LHP-B** were synthesized following a similar procedure. The injection of 1 ml of cesium carbonate solution was performed at 187 °C. The crude solution was centrifuged at 12100 rpm for 5 minutes. The precipitate was dispersed in 1.2 ml of toluene, centrifuged again at 10000 rpm for 3 minutes, and the supernatant was collected.

For the synthesis of $CsPbBr_3$ NCs **LHP-C**, 0.9 ml of OAc and 0.9 ml of OAm were injected at 125 °C, while 1 ml of cesium carbonate solution was injected at 193 °C. The crude solution was centrifuged at 11400 rpm for 5 minutes. The precipitate was dispersed in 1.5 ml of toluene, centrifuged again at 7800 rpm for 3 minutes, and the supernatant was collected.

The synthesis of the $CsPbBr_3$ NCs used in Section 7 of this SI was performed by using: (*i*) a $PbBr_2$-TOPO stock solution (0.04 M) where $PbBr_2$ stock solution was prepared by dissolving $PbBr_2$ (1 mmol, 376 mg) and TOPO (5 mmol, 2.15 g) in octane (5 mL) at 100 ºC, followed by dilution with hexane (20 mL) and filtering through a 0.2 μL PTFE filter before use; (*ii*) a Cs-dopa stock solution (0.02M) that was prepared by mixing 100 mg of Cs_2CO_3 together with 1 ml of diisooctylphosphinic acid and octane (2 mL) at 100 ºC, followed by dilution with hexane (27 mL) and filtering through a 0.2 μL PTFE filter before use; (*iii*) a 1,2-Dioleoyl-sn-glycero-3-

phosphoethanolamine (OA_2-PEA) stock solution (0.1 M) that was prepared by dissolving 74 mg of OA_2-PEA in 1 mL anhydrous toluene. The synthesis protocol (quite similar to the one adopted in Ref.[2]) was the following: in a 25-ml one-neck flask, 2.2 mL $PbBr_2$-TOPO stock solution was combined with 3 mL hexane. Under vigorous stirring, 1 mL of Cs-dopa stock solution was swiftly injected. In 2 min 30s, a stock solution of ligands (94 mL OA_2-PEA in toluene) is added to initiate the ligand exchange on the NC surface. 2 min after the addition of ligands, the crude solution was concentrated by evaporating hexane on a rotary evaporator down to less than 0.5 ml of residual solvent. The NCs were purified using an ethyl acetate and acetonitrile mixture (2:1, v/v), followed by centrifugation and solubilization of the obtained precipitate of NCs in 2 mL cyclohexane.

2 – Analysis of the TEM images

TEM images were collected using a JEOL JEM-2200FS microscope operated at 200 kV. The analysis of the TEM images (column 1 of **Figure S1**) of the three samples listed in Table 1 of the main text was carried out as follows.

The TEM images were analyzed with ImageJ to identify the NCs. A bandpass filter was applied to remove both the speckle pattern typical of the acquisition and the long-ranged modulations due to the inhomogeneous illumination, allowing to apply a threshold to the image. The NCs were then recognized by the Trackmate plugin[3] (thresholding detection), with suitable filters applied and some manual refinements made. This plugin provides both the area and the center of mass of the nanocrystals. Next, from the area A_i of each NC we calculated the NC edge length $\ell_i = \sqrt{A_i}$. The ℓ_i values were gathered in the N_{bin} bins of histograms $P_n(\ell_i)$ reported in column 2 of Figure S1 and the $n-$ and $z-$averages of ℓ along with variances were computed as

$$\langle \ell \rangle_n = \frac{\sum_{i=1}^{N_{bin}} \ell_i \, P_n(\ell_i)}{\sum_{i=1}^{N_{bin}} P_n(\ell_i)} \qquad \sigma_n^2 = \frac{\sum_{i=1}^{N_{bin}} (\ell_i - \langle \ell \rangle_n)^2 \, P_n(\ell_i)}{\sum_{i=1}^{N_{bin}} P_n(\ell_i)}$$

$$\langle \ell \rangle_z = \frac{\sum_{i=1}^{N_{bin}} \ell_i \, P_z(\ell_i)}{\sum_{i=1}^{N_{bin}} P_z(\ell_i)} \qquad \sigma_z^2 = \frac{\sum_{i=1}^{N_{bin}} (\ell_i - \langle \ell \rangle_z)^2 \, P_z(\ell_i)}{\sum_{i=1}^{N_{bin}} P_z(\ell_i)} \qquad \text{(S1)}$$

where $P_z(\ell_i) = (\ell_i)^6 P_n(\ell_i)$.

The $n-$average edge length $\langle L \rangle_n$ of the whole (core +ligand shell) NCs was determined by recovering the histograms $P_n(d_i)$ of the distances between all the NCs centers of mass (column 3 of Figure S1). By assuming that (*i*) the NC are in contact with no ligands overlapping, (*ii*) they are cubic in shape, and (*iii*) they are arranged on a square periodic grid with one face aligned parallel to the substrate, the length $\langle L \rangle_n$ can be determined as the average distance to the nearest neighboring NC, which relates to the first (left) peak of the histograms $P_n(d_i)$.

By comparing $\langle L \rangle_n$ and $\langle \ell \rangle_n$ we recovered the average effective ligand shell thickness $\langle a \rangle$:

$$\langle a \rangle = \frac{1}{2}[\langle L \rangle_n - \langle \ell \rangle_n] \qquad (S2)$$

Finally, we estimated $\langle L \rangle_z$ as

$$\langle L \rangle_z = \langle \ell \rangle_z + 2\langle a \rangle \qquad \text{(S3)}$$

which is the quantity that compares directly with the DLS estimate $\langle L_{\mathrm{DLS}} \rangle_z$ (see Table S1).

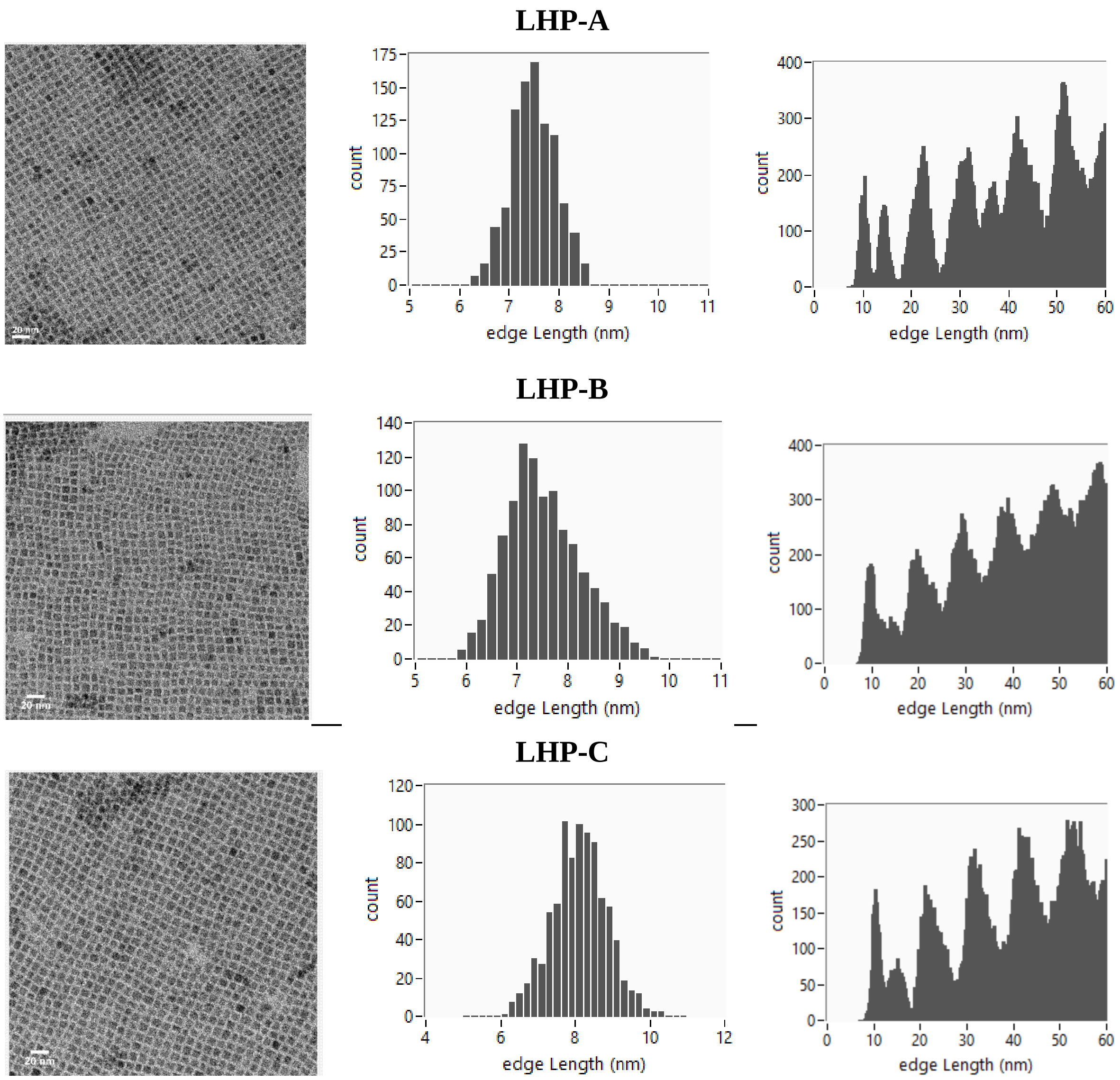

Figure S1: TEM images and analysis for the three NC samples listed in Table 1 of the main text. Left column: raw TEM images; central column: histogram $P_n(\ell_i)$ of the core edge length size; right column: histogram $P_n(d_i)$ of the distances between all the NCs centers of mass.

Table S1: Results from TEM analysis

	core			ligands	core + shell	
Sample	$\langle \ell_{\mathrm{TEM}} \rangle_n$ (nm)	$\frac{\sigma_n}{\langle \ell \rangle_n}$	$\langle \ell_{\mathrm{TEM}} \rangle_z$ (nm)	$\langle a_{\mathrm{TEM}} \rangle$ (nm)	$\langle L_{\mathrm{TEM}} \rangle_n$ (nm)	$\langle L_{\mathrm{TEM}} \rangle_z$ (nm)
LHP-A	7.47	0.06	7.63	1.32	10.12	10.28
LHP-B	7.50	0.10	7.93	1.11	9.71	10.14
LHP-C	8.09	0.08	8.47	1.17	10.43	10.81

3 – Sizing curves from ABS measurements

Here we report a comparison among the various sizing curves available in literature that permit to estimate the NCs edge length $\langle \ell \rangle_n$ from the energy gap E_g extracted from the Absorbance measurements. The black one (Aubert et al.[4]) is the one used in this work, as motivated in the main text. Note that for the sizes of the colloidal nanocrystals studied in this work ($\sim 7 - 8$ nm), a large spread of energy gap values (and viceversa) is visible, making the use of experimentally determined curves rather questionable.

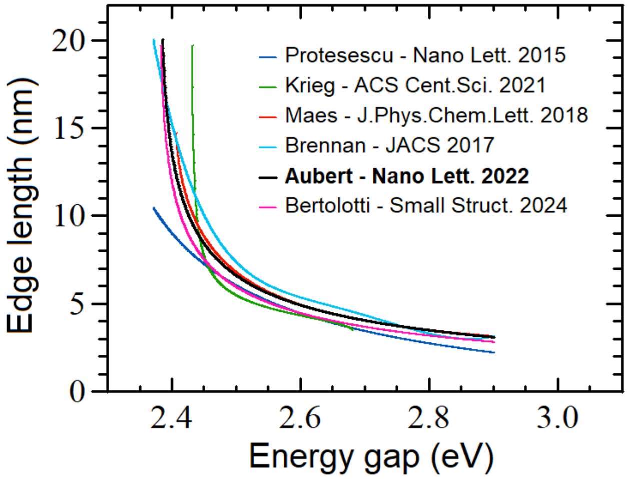

Figure S2: Comparison among various sizing curves available in literature.

4 – Aggregation kinetics of all the OAc/OAm stabilized $CsPbBr_3$ perovskite NCs

The analysis carried out on sample LHP_A of Table 1 of the main text diluted 1:100, was conducted on all three samples of Table 1 at three different dilutions, namely 1:50, 1:100, 1:200. For checking reproducibility, some specific samples at the same dilutions were measured twice. They are indicated below with labels "(1)" and "(2)".

The overall DLS results are illustrated in **Figure S3**, where the error bars on $\langle d_2 \rangle_z$ indicate the standard deviations σ_{d_2} of the corresponding LogNormal distributions (and *not* the parameter uncertainties). Notice that we applied the morphological shape correction [see Eq.(5)] only to the (monodisperse) NCs. Overall, the behaviors of all the samples are rather similar, with the presence in solution of a remarkably small fraction of large micro-sized highly polydisperse aggregates and a large number of small NCs. Although the latter ones are quite difficult to characterize with DLS, they exhibit a slight tendency to increase their sizes, a trend which is much more evident in the ABS and PL data later shown.

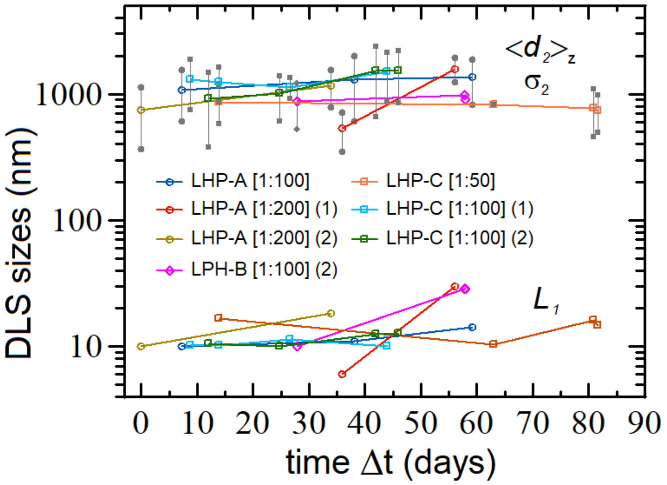

Figure S3: Results extracted from the analysis of DLS data taken during a time interval $\Delta t \sim 7 - 80$ days for all the dilutions (quoted inside square parenthesis) of all the samples of Table 1. $\langle d_2 \rangle_z$ and σ_{d_2} indicate the average hydrodynamic diameter e corresponding standard deviations of the aggregates, whereas L_1 is the edge length of the individual (monodisperse) NCs.

The overall ABS results are illustrated in **Figure S4a**, where we report the time evolution of the wavelength λ_g, corresponding to the energy gap of the first electronic transition. Whereas there are some clear differences between the three samples (see for example the initial values), λ_g systematically increases with time, at faster rates for more diluted conditions. The largest shift in λ_g ($\sim$10 nm) was attained after $\Delta t \sim 60 - 80$ days, with no indication of leveling even after 2 months. Associated with the increase of λ_g's, we expect a corresponding increase of edge lengths ℓ_{ABS} of the individual NCs. However, due to the inconsistency of the available $\ell_{\mathrm{ABS}}(E_g)$ calibration curves (see Figure S2), we chose to display in Figure S4b only the relative changes $\Delta\ell_{\mathrm{ABS}}/\ell_{\mathrm{ABS}}(0)$, *which are almost curve independent*. Consistently with Figure S4a, the larger ℓ_{ABS} increases (which can be as high as $\sim 20 - 40\%$) are seen after $\Delta t \sim 60 - 80$ days, for the highest dilutions.

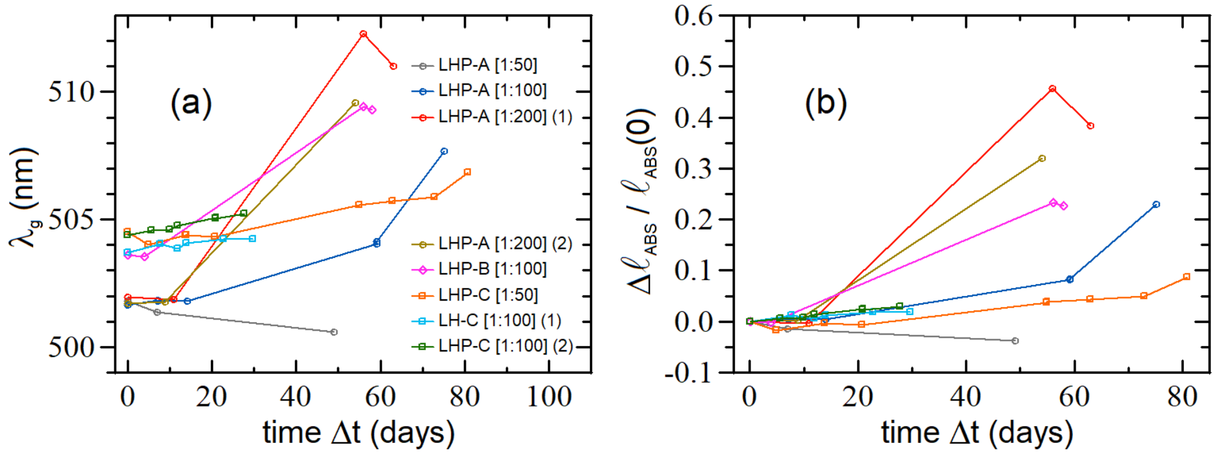

Figure S4: Results extracted from the analysis of ABS data taken during a time interval $\Delta t \sim 7 - 80$ days for all the dilutions of all the samples of Table 1; panel (a): time evolution of the wavelength λ_g corresponding to the energy gap E_g of NCs' the first electronic transition; panel (b): time evolution of the relative increase of the edge length ℓ_{ABS} of individual NCs estimated via the three calibration curves quoted in Table 1.

The overall PL results are illustrated in **Figure S5**, where we report the time evolution of the PL peak wavelength λ_{peak} (panel a) and the PL intensities normalized to their initial values (panel b). Figure S5a is qualitatively similar to Figure S4a, meaning that, for all the three samples, λ_{peak} increases with time, at faster rates for higher dilutions. Similarly, the largest change in λ_{peak} (~10 nm) occurred after $\Delta t \sim 60 - 80$ days, with no sign of reaching a stable state. Finally, Figure S5b shows that all samples exhibit a remarkable reduction of the PL intensities, down to $\sim 10 - 20\%$ of their initial values.

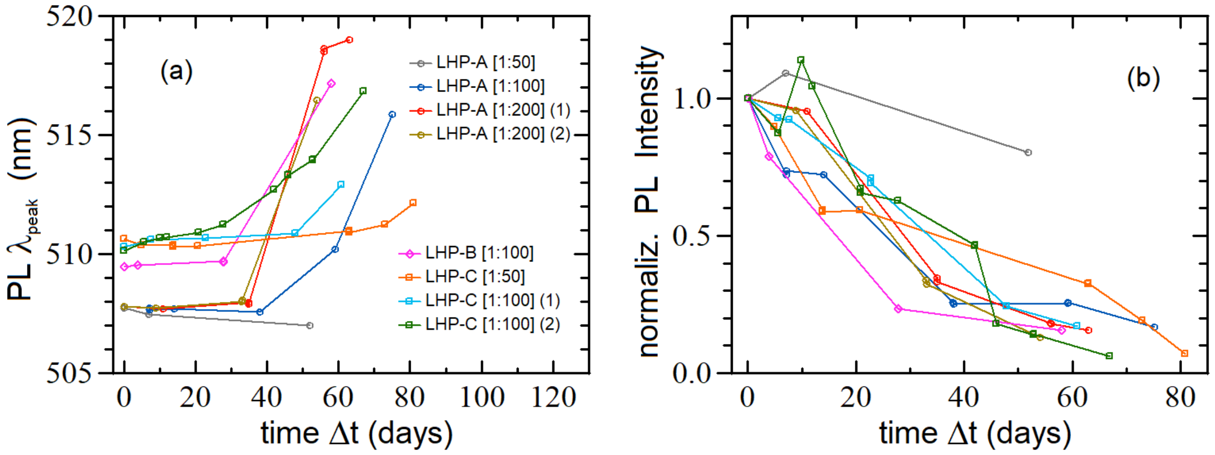

Figure S5: Results extracted from the analysis of PL data taken during a time interval $\Delta t \sim 7 - 80$ days for all three dilutions of samples LHP-A, LHP-B and LHP-C; time evolution, in (a), of the PL peak wavelength λ_{peak}, and, in (b), of the PL intensities normalized to their initial values.

5 – Estimate of the relative scattering and fluorescence signals

The analysis of the DLS data can be fruitfully used for estimating the relative strength between the scattering and fluorescence signals of the pristine (unaggregated) NCs. Indeed, by fitting the data to Eq. (3) of the main text (here reported as Eq. (S4) for the reader's convenience)

$$g_2(q,\tau) = B + \beta_{\mathrm{obs}} \left[\exp\left(-\frac{\tau}{\tau_c(q)}\right)\right]^2 \tag{S4}$$

we can recover (among the others) the parameter, β_{obs} that reads

$$\beta_{\mathrm{obs}} = \beta_{\mathrm{ca}} \frac{\langle I_{\mathrm{sca}} \rangle^2}{\langle I_{\mathrm{tot}} \rangle^2} \tag{S5}$$

where β_{ca} is the spatial coherence factor, $\langle I_{\mathrm{sca}} \rangle$ is the average scattered intensity and $\langle I_{\mathrm{tot}} \rangle$ is the average total detected intensity given by $\langle I_{\mathrm{tot}} \rangle = \langle I_{\mathrm{sca}} \rangle + \langle I_{\mathrm{bkg}} \rangle$, being $\langle I_{\mathrm{bkg}} \rangle$ a background contribution that sums up incoherently to the scattering signal. In our case, since the detection is carried out with a single-mode fiber, β_{ca} = 1 and $\langle I_{\mathrm{bkg}} \rangle = \langle I_{\mathrm{fluo}} \rangle + \langle I_{\mathrm{drk}} \rangle$. Thus, by knowing $\langle I_{\mathrm{tot}} \rangle$ and $\langle I_{\mathrm{drk}} \rangle$ (estimated experimentally) and β_{obs} (recovered from the fitting) it is straightforward to express the ratio α between the scattering and (scattering + fluorescence) signals in terms of known quantities as

$$\alpha \equiv \frac{\langle I_{\mathrm{sca}} \rangle}{\langle I_{\mathrm{sca}} \rangle + \langle I_{\mathrm{fluo}} \rangle} = \sqrt{\beta_{\mathrm{obs}}} \frac{\langle I_{\mathrm{tot}} \rangle}{\langle I_{\mathrm{tot}} \rangle - \langle I_{\mathrm{drk}} \rangle} \tag{S6}$$

Since typically the ratio $\langle I_{\mathrm{drk}} \rangle / \langle I_{\mathrm{tot}} \rangle \ll 1$, α is expected to be slightly larger than $\sqrt{\beta_{\mathrm{obs}}}$.
In our case, where the DLS data were taken with the green laser ($\lambda = 532$ nm), $\langle I_{\mathrm{drk}} \rangle \sim 0.25$ kHz and, for the pristine (unaggregated) NC sample studied in the main text (sample LHP-A of Table 1), we have $\langle I_{\mathrm{tot}} \rangle \sim 1.5$ kHz (see Sect. 4.1 and Figure 4a). By fitting the data corresponding to this sample we estimated $\beta_{\mathrm{obs}} = 0.13$ and by using Eq. (S6), we obtained $\alpha = 0.43$. Thus, about 40% of the (dark subtracted) signal is due to scattering.

6 – Comparison between DLS data taken with green and red lasers

Our setup offers the opportunity of using three lasers operating at different wavelengths, namely a blue (405 nm), a green (532 nm) and a red laser (638 nm). By excluding the blue laser whose wavelength is well inside the NCs absorption band, the green and the red lasers appear to be rather competitive as far as the DLS measurement is concerned. Since the red laser does not excite any fluorescence in our NCs, whereas the green laser (having a photon energy close to the rising edge of the absorption band) make NCs fluoresce, the former may appear more suitable to avoid DLS signal contamination. However, there are several factors pointing at the use of the green laser: (i) its power (100 mW) is higher than that of the red one (50 mW); (ii) the photodetectors quantum efficiency is approximately 2.7 times greater in the green (than in the red) region of the spectrum; (iii) additionally, since the scattering efficiency of the NCs scales as λ^{-4} (Rayleigh scattering), a further factor of $(638/532)^4$~ 2.1 favors the green laser. Taking all these factors into account, it is not clear *a priori* which of the two lasers provides more robust correlations.

To solve this issue, we compare the actual performances of the two lasers by taking DLS measurements on the same perovskite colloidal suspension made of NCs like those reported in Table 1 of the main text. The sample was synthetized at a somewhat higher concentration (~ 20 mg/mL) and diluted 50 × with pure solvent so that the final concentration was ~ 0.4 mg/mL. The DLS data were acquired for the same measuring time ($T_{\mathrm{meas}} = 6000$ s), one after the other, using the two lasers operated at full power. The recovered g_2 curves were nicely fitted with a single exponential decay function (Eq. S4), suggesting that the sample is fairly monodisperse. The results of the two measurements are displayed in **Figure S6** and quantitatively compared in Table S2. As clearly shown in Figure S6a, the correlations from the measurements using both the green and red lasers are accurately reconstructed by the fitting function, without systematic residuals, Figure S6b. Note that, for better readability, we have magnified the error bars of the green data in Figure S6a by a factor of 10. The hydrodynamic diameters recovered from the two data fittings are similar, being $(d_h)_{\mathrm{green}}$ = 16.5 ± 0.1 nm and $(d_h)_{\mathrm{red}}$ = 16.1 ± 0.2 nm.

Figure S6 shows quite clearly that the main difference between the two data sets is their Signal-to-Noise (SNR) ratio. Among the various definitions of SNR that can be adopted for quantifying the noise in DLS data, we use

$$\mathrm{SNR} = \frac{\beta_{\mathrm{obs}}}{\varepsilon_{rms}} \qquad (S7)$$

where $\varepsilon_{rms} = \sqrt{\frac{1}{N^*}\sum_{i=1}^{N^*}\left[g_2^{\mathrm{dat}}(\tau_i) - g_2^{\mathrm{fit}}(\tau_i)\right]^2}$ is the root mean square deviations between data and fit (N^* being the number of lag times with $\tau \leq 10^{-6}$ s). Due to the absence of fluorescence, the red data have a higher intercept β_{obs} but a much higher ε_{rms} due to the weaker scattering signal $\langle I \rangle_{\mathrm{red}} \sim 1.8$ kHz. Conversely, the green data have a lower intercept, but a much smaller ε_{rms} due to the higher scattering signal $\langle I \rangle_{\mathrm{green}} \sim 15$ kHz. The final result is that the SNR is much better for the green laser than for the red laser, as shown in the last column of Table S2.

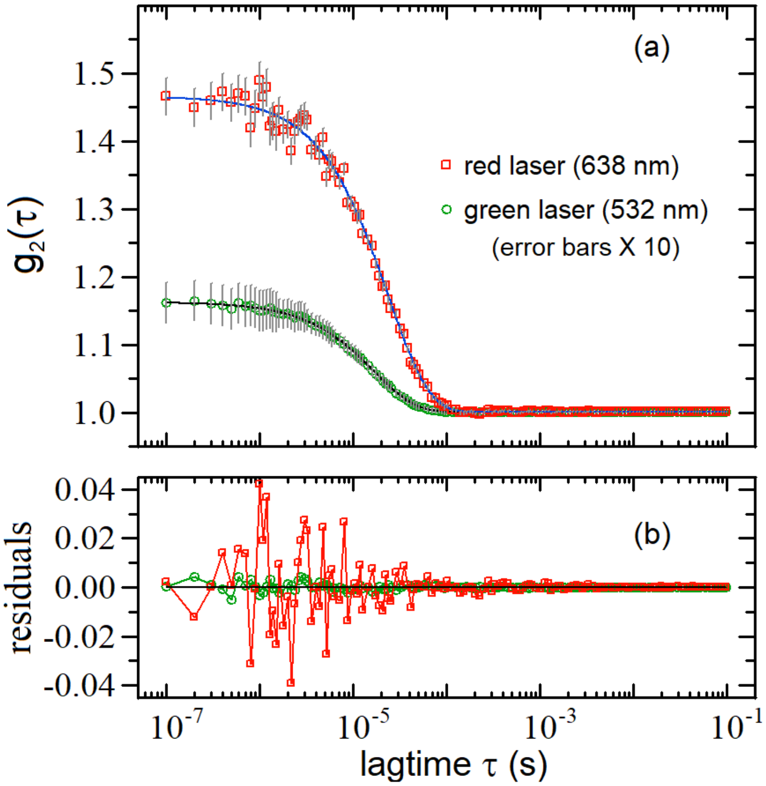

Figure S6 (a): Comparison between DLS data (symbols) taken with a green and a red laser and corresponding fits to Eq. S4 (black curves) for the same NCs perovskite colloidal suspension. For readability, the error bars of the green data have been magnified by a factor 10. Details on measurement parameters are reported in Table S2. (b): relative residuals between data and fit.

Table S2: Comparison between DLS measurements carried out with green and red lasers

	measure parameters				fit results		noise analysis	
Laser	λ (nm)	Power$^{(*)}$ (mW)	$\langle I \rangle$ (kHz)	T_{meas} (s)	d_h (nm)	β_{obs}	ε_{rms}	SNR
green	532	100 (86)	15	6000	16.5 ± 0.1	0.162	3.07×10^{-3}	52.8
red	638	50 (37)	1.8	6000	16.1 ± 0.2	0.465	1.93×10^{-2}	24.1

(*) nominal power and in parenthesis the actual power impinging on the scattering cell.

7 – Comparison between DLS results taken with our setup and commercial instruments.

To check the correct functioning and ascertain the accuracy of our setup, we compared our DLS, ABS and PL measurements with the corresponding results obtained by using commercial instrumentation. For this test we used a recent $CsPbBr_3$ NCs colloidal suspension stabilized via OA_2-PEA ligands[2] (see Sect.1 of this SI for the synthesis). The suspension, originally prepared at ~ 20 mg/mL concentration, was diluted by a factor 200, measured with our setup (green laser) and with: (*i*) a DynaPro (Protein solutions) operating with a laser at $\lambda = 825$ nm) for DLS, (*ii*) a two-arms standard spectrophotometer (Perkin Elmer, model lambda-2 working in the UV-VIS-NIR range) for ABS, and (*iii*) a fiber optics spectrophotometer (Avantes, model AvaSpec-ULS2048x64-EVO) illuminated with a 405nm Laser for PL.

The overall comparison is showcased in **Figure S7**, where the three columns refer to the DLS, ABS, and (normalized) PL data. For ABS (central column, b) and PL (right column, c) we compared directly the data, which exhibit rather small differences, of the order of $\sim 10^{-2}$ or smaller [see panels (b2) and (b3)]. Conversely for DLS (left column), where the $g_2(\tau)$ data are clearly different because of the different wavelengths used in the two instruments, the comparison was carried out by ascertaining the accuracies between data and fits, and comparing the two recovered hydrodynamic diameters. For both data of panel (a1), we successfully used a bimodal fitting function (for taking into account residual large impurities present in the suspension) capable of fitting the data quite accurately, with deviations of the order of $\sim 10^{-3}$ or smaller [see panel (b1)]. The corresponding hydrodynamic diameters recovered were $(d_h)_{\mathrm{our\ setup}}$ = 25.1 ± 0.4 nm and $(d_h)_{\mathrm{DynaPro}}$ = 25.0 ± 0.2 nm.

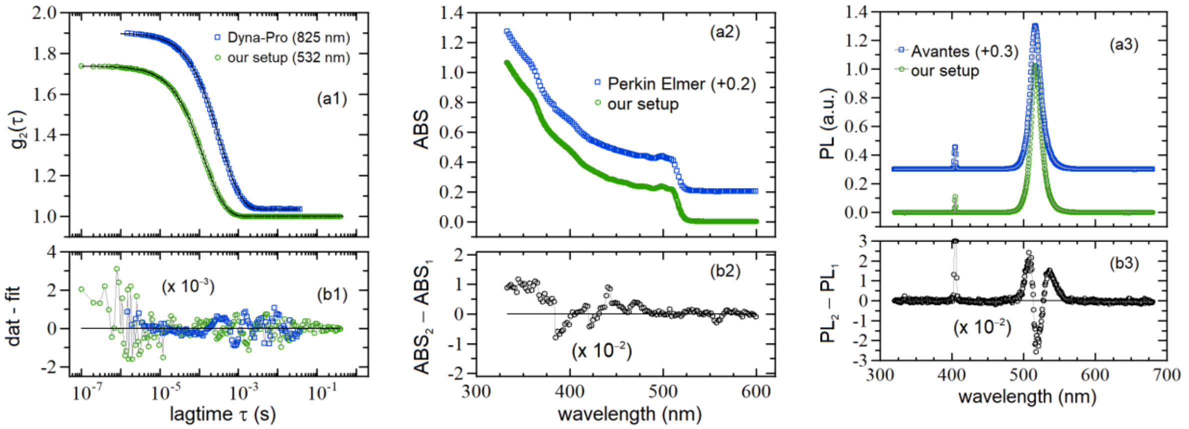

Figure S7. Comparison between DLS (left), ABS (central) and normalized PL (right) data taken with our setup and with different commercial instruments. In the case of DLS (a1) the data fittings are shown as solid black lines. The sample was a perovskite colloidal suspension at a 0.1 mg/mL concentration. For clarity, in panels (a2) and (a3) the blue curves corresponding the commercial instruments have been shifted upwards by the offsets indicated in the labels. Panel (b1) shows the residuals between DLS data and fit, whereas panels (b2) and (b3) the differences between ABS and PL data, respectively. For DLS the deviations are $\sim 10^{-3}$ whereas for ABS and PL are $\sim 10^{-2}$.

8 – Characterization of $CsPbBr_3$ perovskite NCs aggregation by Static Light Scattering

To crosscheck the validity and interpretation of the DLS results reported in the main text, we performed a Static Light Scattering (SLS) characterization of the aggregation kinetics of the same sample described in Figure 4 of Sect. 4.1.1. The SLS measurements were carried out by using an instrument[5] operating at $\lambda = 632.8$ nm, with 18 available fixed scattering angles (15 actually used in this study), covering a q-range of $\sim 2 - 30\ \mu m^{-1}$.

Figure S8 reports the measured scattered intensity distribution $I(q)$ (symbols) and corresponding fittings (solid curves) for a number of times Δt after dilution similar to the ones reported in Figure 4b. Initially, up to a latency time of approximately $\Delta t \sim 7$ h, $I(q)$ is almost flat and constant; later on, it starts to increase and change its shape, with the light being progressively scattered in the forward direction (at low q's). For times longer than $\Delta t = 16$ h, we kept observing a change of the $I(q)$ shape, which became increasingly more and more similar to a straight line, with the slope indicated in the figure. However, due to sedimentation of the large aggregates, the change of the $I(q)$ shape was accompanied by a gradual systematic decrease of the

$I(q)$ amplitude (data not shown for clarity), until $I(q)$ returned (at $\Delta t \geq \sim 20$ h) to the same shape and amplitude of the lowest $I(q)$ reported in Figure S8 ($\Delta t = 0.8$ h).

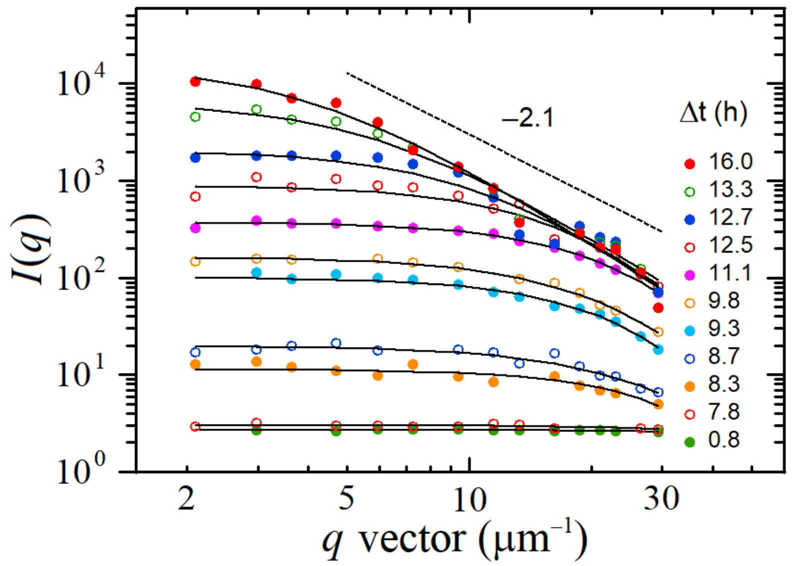

Figure S8: scattered intensity distribution $I(q)$ data (symbols) and fits (continuous lines) for different times after dilution for the same sample described in Figure 4 of the main text. The slope indicates the mass fractal dimension of the NCs aggregates.

The data of Figure S8 were fitted by means of the Fisher-Burford equation[6], which is typically used for describing fractal aggregates

$$I(q) = \frac{I(q=0)}{\left[1 + \frac{2}{3D_{\mathrm{m}}} q^2 R_G^2\right]^{\frac{D_{\mathrm{m}}}{2}}} \qquad \text{(S8)}$$

where the zero-q intensity $I(q = 0)$, the fractal dimension D_{m} and the gyration radius R_G of the aggregates were left as free parameters. Figure S8 shows the typical behavior expected for the evolution of $I(q)$ in a colloidal aggregation experiment: a strong (~ 3.5 decades or larger) increase of the zero-q scattered intensity, accompanied by a pronounced change in the shape of $I(q)$. Specifically, the roll-off of the curves move progressively towards low q's whereas the large-q data collapse on the same asymptote. The latter feature is the signature of the aggregates fractal

morphology, and represents a measure of their mass fractal dimension D_{m} because asymptotically $(q \rightarrow \infty) \rightarrow I(q) \sim q^{-D_{\mathrm{m}}}$. The fittings, reported in Figure S7 as solid curves, are quite satisfactory and allowed to estimate $D_{\mathrm{m}} = 2.1 \pm 0.1$, a figure that is consistent with a Reaction Limited Cluster Aggregation (RLCA) growth modality.[7] At the same time we were able to estimate the increase of R_G, which passed from the a value of $R_G \sim 137$ nm μm at $\Delta t = 8.2$ h. to $R_G \sim 1$ μm at $\Delta t = 16$ h. Assuming that the aggregates behave as compact round objects and recalling that, for a sphere, the equivalent hydrodynamic diameter is $d_h = 2\sqrt{5/3}\, R_G$, the estimated increase of d_h is from $d_h \sim 354$ nm to $d_h \sim 2.5$ μm. Data taken at $\Delta t < 8.2$ h could not be fitted reliably and therefore we did not provide any estimate of R_G.

We conclude by emphasizing that, although subjected to the limitations imposed by sample reproducibility thoroughly discussed in the main text, the entire evolution kinetics observed with SLS is qualitatively consistent with the DLS results obtained with our setup, including also the estimated latency and settling times.

9 – Estimation of noise associated to DLS data and corresponding detection limits

A quantification of the noise associated to DLS data is not straightforward[8,9]. However, under standard experimental conditions, the noise on the first channels of the correlation function is dominated by the shot noise. This occurs because the average number of photo counts $< n >$ on the first lag-times is $< n > = < I > \Delta t_0 \ll 1$, where $< I >$ is the average count rate and Δt_0 is the gate times of the first 16 lag-times τ of $\sigma_{g_2}(\tau)$. This condition is always fulfilled in all our measurements because $\Delta t_0 = 10^{-7}$ s and, even at the highest count rate, we have $< I > \leq 5 \times 10^4$Hz. Thus, the standard deviation associated to the correlation function in the limit of zero lag-times can be estimated as [8,9]

$$\sigma_{g_2}(\tau \rightarrow 0) \sim \frac{\sqrt{1 + \beta_{\mathrm{obs}}}}{< n > \sqrt{M}} \qquad \text{(S9)}$$

where $M = T_{\mathrm{meas}}/\Delta t_0$ is the number of acquired gate times. The corresponding signal-to-noise defined as $\mathrm{SNR} \equiv \beta_{\mathrm{obs}}/\sigma_{g_2}$ (see Eq.S7) is

$$\mathrm{SNR}(\tau \to 0) \sim \frac{\beta_{\mathrm{obs}} < n > \sqrt{M}}{\sqrt{1+\beta_{\mathrm{obs}}}} \tag{S10}$$

Thus, Eq.(S10) can be used for estimating the measuring time necessary for getting an accurate DLS measurement. For example, for $< I > = 3 \times 10^4$Hz and $\beta_{\mathrm{obs}} = 0.5$, it turns out that for having a $SNR \geq 100$, we should have $M \geq 7 \times 10^9$ or equivalently $T_{\mathrm{meas}} \geq 700$ s.

In summary, unless the sample under investigation and the experimental conditions are exceptionally stable (in which case there is in principle no limitation on T_{meas}), satisfying Eq.(S10) requires a tradeoff between T_{meas} and $< n >$, the latter been depending on laser power, detector efficiency, and sample scattering power.

10 – Top view photo of the actual instrumental setup

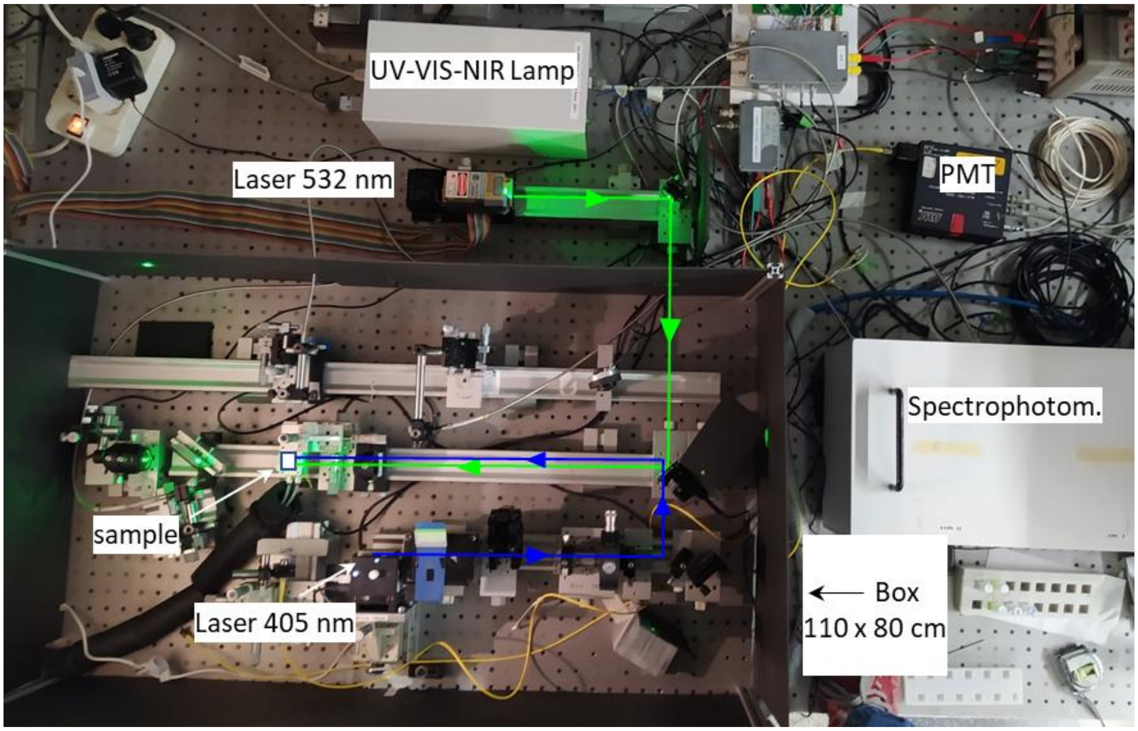

Figure S9: top view photo of the actual instrumental setup. The light sensitive components of the setup are kept inside a 100 x 60 cm box, which can be obscured. The rest of the components are placed on an optical table, including the 638 nm laser not shown in the picture.

References

(1) Shynkarenko, Y.; Bodnarchuk, M. I.; Bernasconi, C.; Berezovska, Y.; Verteletskyi, V.; Ochsenbein, S. T.; Kovalenko, M. V. Direct Synthesis of Quaternary Alkylammonium-Capped Perovskite Nanocrystals for Efficient Blue and Green Light-Emitting Diodes. *ACS Energy Lett.* **2019**, *4* (11), 2703–2711. https://doi.org/10.1021/acsenergylett.9b01915.

(2) Akkerman, Q. A.; Nguyen, T. P. T.; Boehme, S. C.; Montanarella, F.; Dirin, D. N.; Wechsler, P.; Beiglböck, F.; Rainò, G.; Erni, R.; Katan, C.; Even, J.; Kovalenko, M. V. Controlling the Nucleation and Growth Kinetics of Lead Halide Perovskite Quantum Dots. *Science* **2022**, *377* (6613), 1406–1412. https://doi.org/10.1126/science.abq3616.

(3) Ershov, D.; Phan, M.-S.; Pylvänäinen, J. W.; Rigaud, S. U.; Le Blanc, L.; Charles-Orszag, A.; Conway, J. R. W.; Laine, R. F.; Roy, N. H.; Bonazzi, D.; Duménil, G.; Jacquemet, G.; Tinevez, J.-Y. TrackMate 7: Integrating State-of-the-Art Segmentation Algorithms into Tracking Pipelines. *Nat Methods* **2022**, *19* (7), 829–832. https://doi.org/10.1038/s41592-022-01507-1.

(4) Aubert, T.; Golovatenko, A. A.; Samoli, M.; Lermusiaux, L.; Zinn, T.; Abécassis, B.; Rodina, A. V.; Hens, Z. General Expression for the Size-Dependent Optical Properties of Quantum Dots. *Nano Lett.* **2022**, *22* (4), 1778–1785. https://doi.org/10.1021/acs.nanolett.2c00056.

(5) Haller, H. R.; Destor, C.; Cannell, D. S. Photometer for Quasielastic and Classical Light Scattering. *Rev. Sci. Instrum.* **1983**, *54* (8), 973–983. https://doi.org/10.1063/1.1137512.

(6) Fisher, M. E.; Burford, R. J. Theory of Critical-Point Scattering and Correlations. I. The Ising Model. *Phys. Rev.* **1967**, *156* (2), 583–622. https://doi.org/10.1103/PhysRev.156.583.

(7) Anzini, P.; Redoglio, D.; Rocco, M.; Masciocchi, N.; Ferri, F. Light Scattering and Turbidimetry Techniques for the Characterization of Nanoparticles and Nanostructured Networks. *Nanomaterials* **2022**, *12* (13), 2214. https://doi.org/10.3390/nano12132214.

(8) Schatzel, K. Noise on Photon Correlation Data. I. Autocorrelation Functions. *Quantum Opt.* **1990**, *2* (4), 287. https://doi.org/10.1088/0954-8998/2/4/002.

(9) Biganzoli, D.; Ferri, F. Statistical Analysis of Dynamic Light Scattering Data: Revisiting and beyond the Schätzel Formulas. *Opt Express* **2018**, *26* (22), 29375–29392. https://doi.org/10.1364/OE.26.029375.
